# Transcriptomics-based anti-tuberculous mechanism of traditional Chinese polyherbal preparation NiuBeiXiaoHe intermediates

**DOI:** 10.3389/fphar.2024.1415951

**Published:** 2024-09-19

**Authors:** Nan Wang, Qianqian Ma, Junxian Zhang, Jie Wang, Xiaojun Li, Yan Liang, Xueqiong Wu

**Affiliations:** ^1^ Tuberculosis Prevention and Control Key Laboratory, Beijing Key Laboratory of New Techniques of Tuberculosis Diagnosis and Treatment, Department of Tuberculosis, The Eighth Medical Center of PLA General Hospital, PLA General Hospital, Beijing, China; ^2^ Graduate School, Hebei North University, Zhangjiakou, Hebei, China

**Keywords:** tuberculosis, Chinese traditional medicine, NiuBeiXiaoHe, transcriptome, mechanism of action

## Abstract

**Background:**

Integrated traditional Chinese medicine and biomedicine is an effective method to treat tuberculosis (TB). In our previous research, traditional Chinese medicine preparation NiuBeiXiaoHe (NBXH) achieved obvious anti-TB effects in animal experiments and clinical practice. However, the action mechanism of NBXH has not been elucidated.

**Method:**

Peripheral blood mononuclear cells (PBMCs) were collected to extract mRNA and differentially expressed (DE) genes were obtained using gene microarray technology. Finally, GEO databases and RT-qPCR were used to verify the results of expression profile.

**Result:**

After MTB infection, most upregulated DE genes in mice were immune-related genes, including *cxcl9*, *camp*, *cfb*, *c4b*, *serpina3g*, and *ngp*. Downregulated DE genes included *lrrc74b*, *sult1d1*, *cxxc4*, and *grip2*. After treatment with NBXH, especially high-dose NBXH, the abnormal gene expression was significantly corrected. Some DE genes have been confirmed in multiple GEO datasets or in pulmonary TB patients through RT-qPCR.

**Conclusion:**

MTB infection led to extensive changes in host gene expression and mainly caused the host’s anti-TB immune responses. The treatment using high-dose NBXH partially repaired the abnormal gene expression, further enhanced the anti-TB immunity included autophagy and NK cell-mediated cytotoxicity, and had a certain inhibitory effect on overactivated immune responses.

## 1 Introduction

Tuberculosis (TB) epidemic is still a serious public health problem. According to the WHO Global Tuberculosis Report 2023, there were about 10.6 million new TB patients, 1.3 million deaths from TB, 410,000 rifampicin-resistant TB (RR-TB), and the burden of RR-TB and multidrug-resistant TB (MDR-TB) continues to increase, with a low global success rate of RR/MDR-TB treatment (only 63%) worldwide in 2022 (Global Tuberculosis Report, 2023). At present, there are many problems in TB treatment: 1) The current anti-TB chemotherapy needs to take a long time, and these long and complex chemotherapy regimens are a severe challenge for TB patients and the public health system. 2) The bacteria have special regulations and drug resistance mechanisms on the intervention of antibiotics, which leads to the gradual increase of drug resistance of *Mycobacterium tuberculosis* (MTB) and needs more expensive and intolerable drugs used for longer treatment (Pontali et al., 2019; Arora et al., 2021). In addition, new drug research and development also need a long time. 3) Anti-TB drugs have great toxic and side effects. Long-term chemotherapy has different degrees of damage to liver function, renal function, gastrointestinal tract, etc*.*, especially about 10% of TB patients have severe liver damage and have to stop chemotherapy (An et al., 2013); 4) The onset and development of TB are closely related to the deficiency of immune function, imbalance of Th1/Th2 immune response, and low immunity (Cai et al., 2019; An et al., 2022). Anti-TB treatment is facing great challenges. Therefore, it is extremely urgent to develop new therapeutic agents, explore new therapeutic approaches, and establish new joint anti-TB treatment programs.

Traditional Chinese medicine (TCM) has been used to treat TB for more than 2000 years, which was recorded in the Internal Classic, the Miraculous Book of Ten Prescriptions, the Medical Biography, etc., Before the invention of chemotherapeutic drugs, TCM played an extremely important role in the treatment and control of TB and accumulated rich clinical experience. It was found that the deficiency of Vital Energy was an important basis for the onset of pulmonary TB, and the treatment principle of “tonifying deficiency and killing insects” was established, which emphasized adjusting the internal factors of the human body and enhancing self-repair ability to achieve the goal of cure TB. TCM has the advantage of comprehensive treatment of TB with multi-components, multi-targets, and multi-systems. Therefore, the auxiliary treatment of pulmonary TB with TCM can build up healthful vital energy, improve the patient’s immunity, repair the damage, cooperate with biomedicine to play an anti-TB role, to improve the patient’s symptoms of TB poisoning, promote the absorption of lesions and the closure of the cavity, and accelerate the negative conversion of sputum bacteria, thus improving the clinical treatment effect (Li et al., 2020). In recent years, modern experimental methods have been used to clarify the material basis of the efficacy of many TCM prescriptions for the treatment of TB, which has promoted the modernization process of TCM prescriptions. A variety of new anti-TB Chinese polyherbal preparations (such as JieHeWan, FeiTai capsule, QinJiaLiFei capsule, etc.,) have obtained new drug certificates approved by the National Medical Products Administration in China.

The TCM compound NiuBeiXiaoHe (NBXH) is an effective anti-TB empirical formula that has been used for a long time in clinical practice, mainly composed of six traditional Chinese medicines, including Fritillaria cirrhosa, Bletilla striata, Houttuynia cordata, Platycodon grandiflorum, Fructus arctii, and glutinous rice. Among them, Fritillaria cirrhosa and Bletilla striata are used as monarch medicine. Fritillaria cirrhosa has the effects of clearing heat and resolving phlegm, moistening the lungs and relieving cough, dispersing nodules and eliminating carbuncle, and is used to relieve cough, expectorate, relieve asthma, inhibit bacteria and inflammation, and widely used in the clinical treatment of respiratory diseases (Wang et al., 2016; Chen et al., 2020; Zhang et al., 2020). Bletilla striata has the effects of astringent hemostasis, dispersing swelling and engendering flesh, clearing heat and removing dampness, and is used to stanch bleeding, resist pathogenic microorganisms, inhibit inflammation, regulate immunity, and used for anti-fibrosis, anti-oxidation, etc., (Jiang et al., 2019; Zhai et al., 2021; Zhang et al., 2021). Therefore, Bletilla striata is mainly used to treat hematemesis, hemoptysis, pulmonary TB, and various ulcer bleeding in clinics. Houttuynia cordata, Platycodon grandiflorum, and Fructus arctii are all minister medicines. Houttuynia cordata has the effects of clearing heat and detoxifying, eliminating carbuncle and discharging pus, promoting diuresis and relieving stranguria, clearing heat and stopping diarrhea, and is used for anti-bacteria, anti-viruses, anti-inflammation, enhancing the immune function, anti-allergy, relieving asthma and cough, etc., (Sekita et al., 2016; Shingnaisui et al., 2018; Utaiwat et al., 2021). Platycodon grandiflorum has the effect of dispersing lung and eliminating phlegm, facilitating pharynx and discharging pus, and is mainly used for relieving cough and eliminating phlegm, anti-inflammation, anti-oxidation, regulating immunity, protecting liver and kidney, etc., (Choi et al., 2017; Zhang et al., 2018; Li and Yang, 2021). Fructus arctii has the effects of dispersing lung and eliminating phlegm, promoting pharynx and rash, detoxifying and detumescence, moistening intestines and relieving constipation, and is used for anti-bacteria, anti-viruses, anti-inflammation, and liver protection (Gao et al., 2018; Li et al., 2022). Glutinous rice is an assistant medicine, which has the effects of tonifying the middle and replenishing qi, strengthening the spleen and stomach, and stopping sweating. To sum up, the main function of this prescription is to moisten the lung and relieve cough, clear heat and eliminate phlegm, and eliminate carbuncle and discharge pus, which can be used for pulmonary TB and lung carbuncle with yin deficiency and phlegm heat, with symptoms of cough, yellow purulent sputum, hemoptysis, fever or low fever in the afternoon, chest tightness or dry stool. The results of our previous studies showed that the extract of the effective TCM compound NBXH in the clinical treatment of TB could improve the immune function, inhibit the growth of MTB, and reduce the degree of the lesion in the treatment of the mouse TB model (Liang et al., 2017; Duan et al., 2021; Liang et al., 2021). At present, this Chinese medicine compound has been developed into a traditional Chinese polyherbal preparation. The preparation process is different from that of TCM decoction. It is necessary to understand its mechanism of action and clarify its target and regulatory pathway. Therefore, based on previous studies (Liang et al., 2017; Duan et al., 2021; Liang et al., 2021), this study constructed a mouse model of MTB acute infection. With traditional Chinese polyherbal preparations JieHeWan (JHW) powder as a control, we studied the changes in gene transcription expression and regulation pathway after MTB infection and after treatment with traditional Chinese polyherbal preparation NBXH intermediates through gene expression microarray technology, and verified the expression of some genes in TB patients through GEO database and RT-PCR, to clarify the mechanism of "NBXH - TB targets - biomolecular network” regulating the body to play an overall anti-TB role.

## 2 Materials and methods

### 2.1 The preparation of NBXH intermediate

NBXH intermediate (lot number H200828) was produced by Xi’an Xintong Pharmaceutical Research Co., Ltd. (Xi’an, China). The preparation process is shown as follows: Taked 167 g Houttuynia cordata, 208 g Platycodon grandiflorum, and 167 g Fructus arctii, with 10 times the amount of water to boil three times continuously. The first and second times were for 2 h and the third was for 1 h. Combined and filtered the decoctions, then concentrated the filtrate under reduced pressure (60°C–70°C) to a thick paste with a relative density of 1.20–1.25 (60°C), dried under reduced pressure, ground the dry extracts and passed through a 100-mesh sieve, and then mixed it with the powder of 208 g Fritillaria cirrhosa, 312 g Bletilla striata, and 188 g glutinous rice, which directly ground and passed through a 100-mesh sieve.

### 2.2 Experimental design and grouping

**FIGURE 1:**
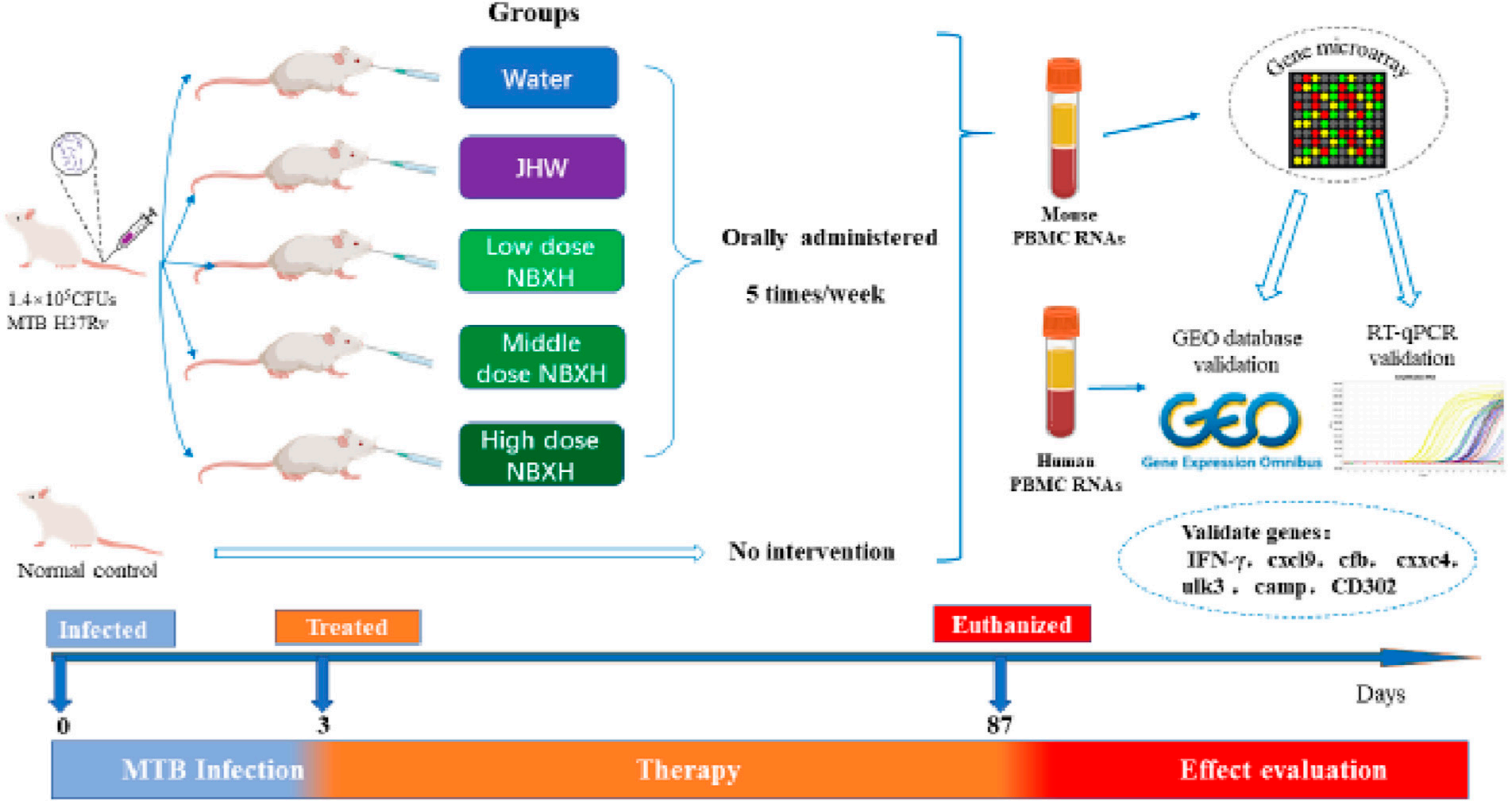
The study flowchart.

Based on the previous animal experimental study on TB treatment with NBXH intermediates (Liang et al., 2017; Duan et al., 2021; Liang et al., 2021), this study further studied the anti-TB action mechanism of NBXH in mice. The complete research design, including animal model construction, grouping, medicine administration plan, gene microarray, and validation of gene expression profile are shown in Figure 1. Briefly, 3 days after MTB infection, 50 mice were randomly divided into the following five groups and orally administered 5 times a week: TB model group (0.5 mL distilled water/day per mouse), JHW treatment group (12 mg/day per mouse), low-dose (2.4 mg/day per mouse) of NBXH intermediate group, middle-dose (4.8 mg/day per mouse) of NBXH intermediate group, and high-dose (9.6 mg/day per mouse) of NBXH intermediate group. On the 87th day after treatment, the mice were euthanized and the total RNAs of peripheral blood mononuclear cells (PBMCs) from three mice were extracted to perform gene microarray analysis to obtain differential gene (DE) profile. Finally, the GEO database and RT-qPCR assay were used to validate the reliability of the DE gene profile.

### 2.3 Experimental animals and ethics

Sixty BALB/c 42–62-day-old female mice were purchased from Beijing Vitalriver Experimental Animal Technology Co., LTD., and put into the animal laboratory of the Eighth Medical Center of the PLA General Hospital for adaptive feeding. The mouse model of acute MTB infection was prepared by injecting 1.4 × 10^5^ CFUs MTB H37Rv standard strain (Laboratory of National Institute for Food and Drug Control) suspension through the tail vein. The animal experiments have been approved by the Animal Ethics Committee of the Eighth Medical Center of the PLA General Hospital (309,202,108,101,027). Animal feeding and management strictly follow the animal management and usage regulations of the animal laboratory of the Eighth Medical Center of the PLA General Hospital.

### 2.4 PBMC isolation and RNA extraction

Three weeks after the last immunization, three mice in each group mentioned above were euthanized, and then their blood was collected in heparin sodium anticoagulant tubes. According to the manufacturer’s instructions, PBMCs were isolated with the Mouse PBMC Isolation Kit (Haoyang Biological Products Technology, Tianjin, China), and total RNA was extracted from PBMCs using TRIzol Reagent (Invitrogen, United States).

### 2.5 Gene microarray expression analysis

The gene microarray experiments were conducted by Kangcheng Biotechnology Co., LTD. (Shanghai, China) using the Agilent Array platform. In brief, RNA samples were identified using a NanoDrop ND-1000 ultraviolet spectrophotometer (Implen, Germany) and agarose gel electrophoresis. Then, according to the experimental instructions of Agilent One-Color Microarray-Based Gene Expression Analysis (Agilent Technology, United States), RNA samples were labeled using Agilent Quick Amp Labeling kit, and hybridization experiments were performed using Agilent mouse 4 × 44 K gene expression profile chip v2 (4 × 44 K, Agilent, United States). The hybrid chip was washed, fixed, and scanned using the Agilent DNA Microarray Scanner (part number G2505°C). Agilent Feature Extraction software (v11.0.1.1) was used to collect the chip probe signal value to obtain the original data. Finally, the GeneSpring GX v12.1 software (Agilent Technology) was used for Quantile standardization and subsequent processing of the original data.

### 2.6 DE genes screening and identification

The DE genes in the TB model group vs. normal group, JHW group vs. TB model group, as well as low, middle, and high dose NBXH groups vs. TB model group were screened by analyzing hierarchical clustering heatmaps, scatter plots, and volcano plots. The Hierarchical cluster plot showed the differences in gene expression among different groups of samples. The scatter plot showed the DE gene correlation between two groups of samples, where the *x*-axis values and the *y*-axis values were the results of mean normalization processing (log2 scaling). The volcano plot showed the distribution of DE genes between two groups of samples, with the *x*-axis representing the log2 value (Fold Change) and the *y*-axis representing the -log10 value (*p*-value).

### 2.7 GO analysis and KEGG analysis

Gene Ontology (GO) is an internationally standardized gene functional classification system that comprehensively describes the attributes of any biological gene and its products by using a dynamically updated controlled vocabulary and strictly defined concepts. In this study, significant DE genes between different groups were subjected to GO term enrichment analysis on biological process (BP), cell component (CC), and molecular function (MF) through the standard vocabulary provided by Gene Ontology (http://www.geneontology.org). to calculate the hypergeometric distribution relationship between DE genes and several specific branches in the GO classification to explore which gene function changes may be related to DE genes in different samples. The Kyoto Encyclopedia of Genes and Genomes (KEGG) database is a database that systematically analyzes gene function, and links genomic information and functional information, and the metabolic pathway database is the most widely used. Through the pathway analysis of DE genes, the metabolic pathways enriched in DE genes were found, and which metabolic pathways might lead to differential gene expression between different groups of samples were identified. The GO and pathway analysis used hypergeometric tests to calculate *p*-values that indicate the importance of the conditional pathway or GO item enrichment in the DE gene. The lower the *p*-value, the more significant the GO term or pathway is (recommended cutoff value 0.05).

### 2.8 GEO database verification

The relevant transcriptome data were downloaded from the GEO database of the National Center for Biotechnology Information (NCBI) (http://www.ncbi.nlm.nih.gov/geo/). A total of 12,505 studies were retrieved using the keyword “Tuberculosis”. Select the GSE dataset with the study type of expression profiling by array, organisms of mouse and *Homo sapiens*, and sample of whole blood or PBMC. The GEO2R tool was used to screen DE genes with the threshold of ∣log2FC∣> 0.5 and adjust the *p-value* < 0.05. Select the datasets associated with TB diagnosis to screen DE genes compared with the top 10 upregulated or downregulated DE genes in the TB model group vs. the normal group in our profile. Then, select the datasets related to TB treatment to screen DE genes compared with the top 10 DE genes in the high-dose NBXH group vs. the TB model group.

Seven datasets, including GSE54992, GSE48027, GSE98461, GSE83456, GSE62525, GSE34608, and GSE14361, were selected to verify the top 10 upregulated or downregulated DE genes in TB model group vs. normal group. The GSE54992 dataset included nine patients with active tuberculosis (ATB) and six healthy controls (HCs) (Cai et al., 2014). The GSE48027 dataset included 6 MTB-infected mouse models and four uninfected healthy mice (Manca et al., 2013). The GSE98461 dataset included 4 ATB patients and 4 HCs (Pan et al., 2017). The GSE83456 dataset included 45 ATB patients and 61HCs (Blankley et al., 2016). The GSE62525 dataset included 14 ATB patients and 14 HCs (Lee et al., 2016). The GSE34608 dataset included 8 ATB patients and 18 HCs (Maertzdorf et al., 2012). The GSE14316 dataset included 4 MTB-infected mouse models and four uninfected healthy mice (Schreiber et al., 2009).

Four datasets, including GSE54992, GSE62147, GSE48027, and GSE31348, were selected to verify the top 10 upregulated or downregulated DE genes in the high-dose NBXH group vs. TB model group. The GSE54992 dataset included 9 ATB patients before and after 26 weeks of treatment (Cai et al., 2014). The GSE62147 dataset included 26 ATB patients before and after 26 weeks of treatment (Tientcheu et al., 2015). The GSE48027 dataset included 2 MTB-infected mouse models before and after 26 weeks of treatment (Manca et al., 2013). The GSE31348 dataset included 27 ATB patients before and after 26 weeks of treatment (Cliff et al., 2013).

### 2.9 RT-qPCR validation experiment

About 10 mL of whole blood from 40 initially treated pulmonary TB patients and 39 HCs was collected in heparin sodium anticoagulant tubes and RNAs were extracted for RT-qPCR assay, to verify the DE genes in the TB model group vs. the normal group. The inclusion criteria of the initially treated pulmonary TB patients (18–60 years old) was sputum-culture positive, and/or Xpert-positive, and/or interferon-γ release assay (IGRA)-positive, without anti-TB treatment or with anti-TB treatment for less than 1 month. Then, PBMCs were respectively isolated from five initially treated pulmonary TB patients before and after chemotherapy combined with NBXH treatment, and RNAs were extracted for RT-qPCR assay to verify the DE gene in the high dose NBXH group vs. TB model group. The inclusion criteria were initial active pulmonary TB patients treated with anti-TB chemotherapy combined with NBXH for ≥8 weeks. All PBMCs were stimulated with CFP10-ESAT6 fusion antigen (Shanghai Jin Nuo, China) for 24 h, all RNAs were extracted using the Trizol reagent (Invitrogen, United States), and 1 μg RNA was reverse-transcribed into cDNA using a reverse transcription kit (Takara, Japan). The quantification of cDNA was examined using RT-qPCR on Roche 480 (Roche, Switzerland) with the following procedure: pre-denatured at 95°C for 3 min; 40 cycles of denaturing at 95°C for 10 s, annealing at 60°C for 20 s; extension at 72°C for 1 s. RT-qPCR of each cDNA sample was repeated twice, and the final Ct value was the mean of the two times. The relative expression levels of all genes were measured by the 2^−△△Ct^ method using GAPDH as the internal reference. The sequences of each gene primer are shown in Table 1.

**TABLE 1:** The primer sequences for amplification of the DE genes.

Gene names	Primer sequences (5´→3′)
*Gapdh*	F: CTC​TGG​TAA​AGT​GGA​TAT​TGT
	R: GGT​GGA​ATC​ATA​TTG​GAA​CA
*Ifn-γ*	F: TCG​GTA​ACT​GAC​TTG​AAT​GTC​CA
	R: TCG​CTT​CCC​TGT​TTT​AGC​TGC
*Cxcl9*	F: CCA​GTA​GTG​AGA​AAG​GGT​CGC
	R: AGG​GCT​TGG​GGC​AAA​TTG​TT
*Cfb*	F: ACG​ACT​TCG​AGA​ACG​GGG​AA
	R: CGT​CAT​AGC​AGT​GGA​AAG​AGA​T
*Cxxc4*	F: TTT​CCG​CTA​TCC​CGG​CTT​TAG
	R: GGC​AAT​TTG​AAA​CGC​ACT​GTC
*Camp*	F: AGG​TCC​TCA​GCT​ACA​AGG​AAG
	R: TCT​TGA​AGT​CAC​AAT​CCT​CTG​GT
*Ulk3*	F: GAA​GGA​CAC​TCG​TGA​AGT​GGT
	R: ACA​ATG​TGG​GGA​TGT​CGA​ATG
*CD302*	F: TGG​AGC​GGA​CAT​GAT​AAG​CAT
	R: TCC​ATT​CAC​CTG​TCT​TGA​TGT​G

### 2.10 Data analysis

The mouse gene expression profile was analyzed by assessing the Fold Change method, and the threshold of Fold Change values for screening upregulated or downregulated DE genes was set to ≥2, with a *p-value* < 0.05. The R language was used for hierarchical cluster analysis. GO analysis and pathway analysis were performed using standard enrichment calculation methods. RT-qPCR data were statistically analyzed using GraphPad Prism nine software (California, United States). The *t*-test was used to compare the relative expression data between ATB patients and HCs, and a *p-value* of <0.05 was considered statistically significant. The relative expression data of ATB patients before and after treatment were used paired *t*-test, and *p-value* < 0.05 was considered to have a statistical difference.

## 3 Result

### 3.1 Anti-TB immune responses in mice infected with MTB

#### 3.1.1 DE genes in the TB model group vs. the normal group

The scatter plot, volcano plot, and cluster diagram of DE genes in the TB model group vs. normal group, JHW group vs. TB model group, and each dose of NBXH group vs. TB model group were shown in Supplementary Figure S1. In the TB model group vs. normal group, the total number of upregulated DE genes was 829, while the total number of downregulated DE genes was 505 (Table 2), indicating that the gene expression in mice was significantly disturbed after MTB infection.

**TABLE 2:** The number of DE genes in TB model group vs. normal group, JHW group vs. TB model group, and each NBXH dose group vs. TB model group.

Number of DE genes	TB model group vs normal group	JHW group vs TB model group	NBXH groups vs TB model group		
			Low dose	Middle dose	High dose
Upregulated expression	829	826	992	433	254
Downregulated expression	505	91	624	352	292

The top 10 upregulated and downregulated DE genes in the TB model group vs. the normal group and their changes in other groups were shown in Tables 3, 4 respectively. The results showed that: 1) In the TB model group vs. normal group, the FC value of the upregulated DE genes was much higher than that of the downregulated DE genes, and the disturbance of gene expression was mainly upregulation. 2) In the TB model group vs. normal group, the upregulated DE genes were all related to host immune defense and cytokine regulation, and the downregulated DE genes involved many functions, including nervous system function regulation, endocrine, immunity, etc., These results indicated that mice infected with MTB mainly upregulated the expression of anti-TB immune-related genes.

**TABLE 3:** The top 10 significantly upregulated DE genes in TB model group vs normal group and their changes in JHW group vs TB model group and each NBXH dose group vs TB model group.

GenBank accession	Gene symbol	TB model group vs normal group	JHW group vs TB model group	NBXH groups vs TB model group			Annotation
				Low dose	Middle dose	High dose	
NM_008599	*Cxcl9*	46↑	no	no	no	3↓	C-X-C Motif Chemokine Ligand 9, also known as an interferon-γ-induced mononuclear factor (MIG), can combine with its receptor CXCR3 to recruit CXCR3^+^ cells, such as monocytes, CD4^+^ Th1 cells, and CD8^+^ cytotoxic T Cells, inducing Th1-type immunity while inhibiting Th2-type immunity and involving in immunoregulatory and inflammatory processes De Benedetti et al. (2021)
NM_009921	*Camp*	35↑	no	no	no	no	Cationic antimicrobial peptide, is one of antimicrobial peptide (AMP) family members, mainly expressed in immune cells. As an endogenous effector molecule in innate immunity, it can directly inhibit pathogen activity and regulate host innate and adaptive immune responses, including inducing pro-inflammatory effects and promoting autophagy Bucki et al. (2010); Bei et al. (2019). It has a broad-spectrum antibacterial activity Dlozi et al. (2022)
NM_008198	*Cfb*	30↑	no	no	no	no	Complement factor B, is an important molecule in the bypass pathway of the complement system and plays an important role in the process of amplifying the activation response of the complement and effectively clearing pathogens. It is closely related to the anti-tuberculosis immune function of the immune system Shi et al. (2022)
NM_009780	*C4b*	26↑	no	no	no	3↓	Complement factor 4B, is involved in the innate immune response and inflammatory response through the classical activation pathway, which is closely related to the anti-tuberculosis immune function of the immune system Daher et al. (2015); Wang et al. (2023)
NM_001034859	*Gm4841*	24↑	no	no	no	4↓	Interferon-inducible GTPase-like protein, predicted to enable GTPase activity, is involved in cellular response to interferon and defense response and is active in endoplasmic reticulum membrane (NCBI)
NM_009251	*Serpina3g*	23↑	no	no	no	2↓	Serpin family A member 3 g, also known as serpin 2A or Spi2A, an intracellular serine-protease inhibitor Salazar-Olivo et al. (2014), participates in multiple immunization activities and plays an important role in regulating cellular processes such as angiogenesis, apoptosis, fibrosis, oxidative stress, and inflammatory response Yuan et al. (2021)
NM_008694	*Ngp*	22↑	no	no	no	no	Neutrophilic granule protein, which belongs to the cystatin superfamily, is an important host defense component that suppresses proinflammatory cytokines (TNF-α, IL-8, and IL-1β) production by targeting NF-κB and promotes anti-inflammatory cytokines (IL-10) secretion, regulating inflammatory responses and enhancing bacterial phagocytosis by activated macrophages Liu et al. (2020)
NM_001101475	*F830016B08Rik*	22↑	no	no	2↓	3↓	-
NM_001033767	*Gm4951*	21↑	no	no	2↓	3↓	Immunity-related GTPase, is transcriptionally induced by IFN-γ and predominantly expressed in hepatocytes. It directly regulates autophagy and plays an important role in the resistance to intracellular pathogens including MTB Pilla-Moffett et al. (2016); Zhang et al. (2022)
NM_001013828	*Iigp1b*	20↑	no	no	no	no	Interferon-inducible GTPase 1b, which belongs to a family of 47-kDa GTPases, is strongly induced transcriptionally by interferons and implicated in cell-autonomous resistance to intracellular pathogens (Uthaiah et al. (2003)

The number below each group means the Fold Change value of DE gene. “↑” means upregulated expression; “↓” means downregulated expression. The following tables of DE, genes all follow this table.

**TABLE 4:** The top 10 significantly downregulated DE genes in TB model group vs normal group and their changes in JHW group vs TB model group and eah NBXH dose group vs TB model group.

GenBank accession	Gene symbol	TB model group vs normal group	JHW group vs TB model group	NBXH groups vs TB model group			Annotation
				Low dose	Middle dose	High dose	
NM_029053	*Lrrc74b*	11↓	no	no	12↑	14↑	-
NM_016771	*Sult1d1*	9↓	no	no	no	no	Sulfotransferase Family 1D Member 1 (Sult1a3 in human), a sulfotransferase, is directly induced by glucocorticoids and may attenuate elevated catecholamine activity during the stress response Wong et al. (2010)
XM_006498495	*Gm2240*	9↓	2↑	no	no	no	-
NM_016766	*Mcrs1*	9↓	no	no	13↑	6↑	Microspherule protein 1, is one of the important cell cycle regulators and participates in various pathways such as regulation of transcription factors for cell proliferation and stress response, histone posttranslational modification, mRNA targeting and translation, telomerase expression, senescence induction, mTOR pathway activation, centrosome integrity, and microtubule dynamics Huang et al., (2023)
NM_029095	*Hhatl*	8↓	no	no	8↑	9↑	Hedgehog acyltransferase, a palmitoylacyltransferase with specificity for N-palmitoylation of Sonic Hedgehog Buglino and Resh (2008). Hedgehog proteins govern crucial developmental steps in animals and drive certain human cancers Jiang et al. (2021)
NM_001004367	*Cxxc4*	8↓	no	no	5↑	10↑	CXXC finger protein 4, is a tumor suppressor regulated by EZH2, functions to inhibit both Wnt/β-catenin signaling pathway by interacting with Disheveled and MAPK/ERK signaling by binding to MEK1, and can stimulate the transcription of GDF15 (growth differentiation factor 15) to activate apoptosis Han et al. (2017) and improve the proliferation of CD3^+^ T Cells through ELK1‐mediated regulation of ERK1/2 axis Li et al. (2020b)
AK052223	*Apol7d*	7↓	no	no	no	no	Apolipoprotein L 7days, a mouse lncRNA gene. Its function is not yet clear (NCBI)
XR_871279	*Gm10030*	7↓	no	no	12↑	10↑	Predicted gene 10,030, a mouse lncRNA gene. Its function is not yet clear (NCBI)
NM_001159507	*Grip2*	7↓	no	no	10↑	no	Glutamate receptor-interacting protein 2, encodes a multi-PDZ-containing protein with an established role in AMPA-receptor trafficking in the neurons and functions downstream of Notch3/RBP-Jκ to regulate myogenic tone in the brain arteries Fouillade et al. (2013). In addition, it is important in the development of innate CD8^+^ T Cells Jo et al. (2020)
NM_001110323	*Klra7*	6↓	no	no	no	5↑	Killer cell lectin-like receptor 7, also known as Ly49 g, has carbohydrate-binding activity, involves the cell adhesion process, and behaves as a general activation marker on the NK cell. The Ly49G^+^ NK cell subset is responsible for mediating tumor killing and critical resistance to mouse viral infection Barao et al. (2011); Barao et al. (2013)

#### 3.1.2 GO analysis in the TB model group vs. the normal group

GO analysis of the top 10 items of CC, BP, and MF in the TB model group vs. the normal group (Supplementary Figure S2) showed that: 1) Upregulated CC items were mainly associated with the extracellular regions, cell surface, and various membrane components, such as cell periphery, cellular anatomical entity, plasma membrane, cell surface, extracellular region, membrane. Downregulated CC items were mainly related to synapses and cell connection components, such as synapse, cell junction, synaptic membrane, neurofilament, postsynapse, and postsynaptic membrane. 2) Upregulated BP items were mainly related to immune defense responses such as defense response, immune system process, response to external stimulus, response to stress, immune response, response to other organisms, response to external biotic stimulus, defense response to other organisms, response to biotic stimulus, interspecies interaction between organisms. Downregulated BP items involved multiple processes such as animal organ morphogenesis, response to steroid hormone, positive regulation of epithelial to mesenchymal transition, cell adhesion, positive regulation of cell differentiation, biological adhesion, water transport. 3) Upregulated MF items mainly involved various substance binding and enzyme activities, such as protein binding, binding, peptide antigen binding, identical protein binding, peptide binding, amide binding, enzyme inhibitor activity, GTPase activity, protein-containing complex binding, signaling receptor binding. Downregulated MF items mainly involved the transporter activity and substance binding, such as inorganic molecular entity transmembrane transporter activity, cation transmembrane transporter activity, carbohydrate binding, transmembrane transporter activity, MHC protein complex binding, ion transmembrane transporter activity, coreceptor activity, water transmembrane transporter activity, transporter activity, and steroid binding.

#### 3.1.3 Pathway enrichment analysis in the TB model group vs. the normal group

**FIGURE 2:**
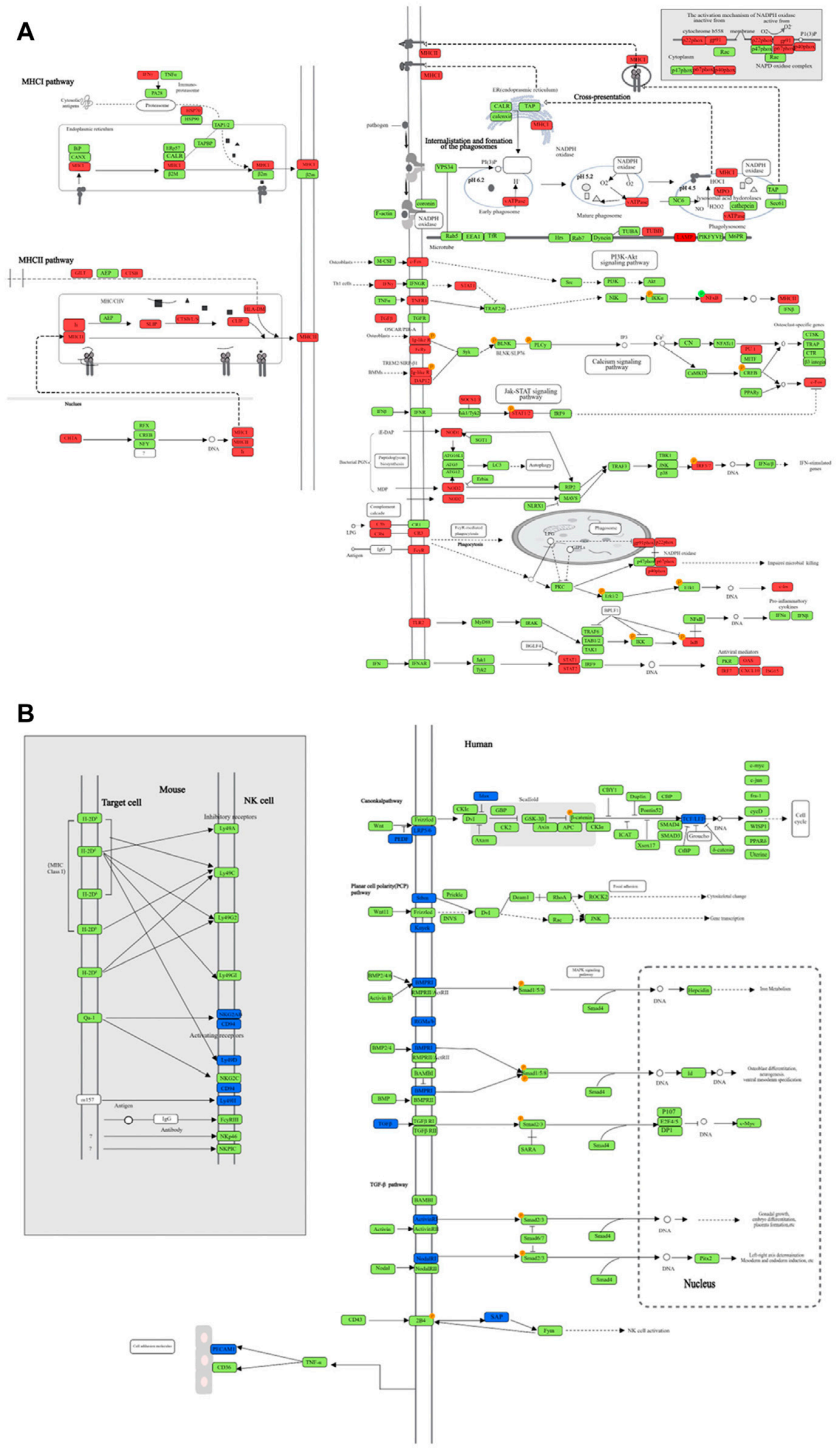
The DE genes in significantly upregulated and downregulated pathways after MTB infection. **(A)** Each pathway shown in Table 5, and the significantly upregulated DE genes in each pathway after MTB infection; **(B)** Each pathway shown in Table 6, and the significantly downregulated DE genes in each pathway after MTB infection. Red represents the upregulated DE genes, green represents unchanged genes, and blue represents downregulated DE genes.

We selected the top 10 upregulated pathways ranked by enrichment score in the TB model group vs. normal group, and then counted the enrichment scores of these pathways in the JHW group vs. TB model group and each dose of NBXH group vs. TB model group (Table 5). Most pathways were related to host immune responses, such as phagosome, antigen processing and presentation, and NOD-like receptor signaling pathway. After summarizing the DE genes involved in each pathway, we found that although these pathways involved various functions, the changed targets in each pathway were certain, mainly related to immune defense responses such as cytokines, complement, and MHC pathways (Figure 2A).

**TABLE 5:** The top 10 significantly upregulated pathways in TB model group and their changes in JHW group and various NBXH groups.

Pathway ID	Definition	TB model group vs. normal group	JHW group vs. TB model group	NBXH groups vs. TB model group		
				Low dose	Middle dose	High dose
mmu04145↑	Phagosome	20.442292↑	NO	NO	NO	3.152111 (↓)
mmu04612↑	Antigen processing and presentation	16.309057↑	NO	NO	NO	4.628266 (↓)
mmu04380↑	Osteoclast differentiation	13.968584↑	NO	NO	NO	NO
mmu05330↑	Allograft rejection	12.022379↑	NO	NO	NO	3.768181 (↓)
mmu05332↑	Graft-versus-host disease	12.022379↑	NO	NO	NO	3.768181 (↓)
mmu05169↑	Epstein-Barr virus infection	11.216051↑	NO	NO	NO	1.458688 (↓)
mmu04940↑	Type I diabetes mellitus	11.048553↑	NO	NO	NO	3.511222 (↓)
mmu05140↑	Leishmaniasis	9.972411↑	NO	NO	NO	NO
mmu05416↑	Viral myocarditis	9.909195↑	NO	NO	NO	2.971572 (↓)
mmu04621↑	NOD-like receptor signaling pathway	9.2449↑	NO	NO	NO	NO

In the TB model group vs. normal group, there were only five downregulated pathways (Table 6), and all the pathways involved were related to host immune responses, such as natural killer cell-mediated cytotoxicity, Wnt signaling pathway, and TGF-β signaling pathway. After summarizing the DE genes involved in the five pathways (Figure 2B), we found that the targets with downregulated differential expression mainly presented in NK cell surface receptors, Wnt signaling pathway, and TGF-β signaling pathway.

**TABLE 6:** The five downregulated pathways in TB model group and their changes in JHW group and various NBXH group.

Pathway ID	Definition	TB model group vs. normal group	JHW vs. TB model	NBXH groups vs. TB model group		
				Low dose	Middle dose	High dose
mmu04650↓	Natural killer cell-mediated cytotoxicity	3.120503↓	NO	NO	NO	5.892759 (↑)
mmu05144↓	Malaria	2.177646↓	NO	NO	NO	3.742157 (↑)
mmu04310↓	Wnt signaling pathway	1.68843↓	NO	NO	NO	2.835092 (↑)
mmu04710↓	Circadian rhythm	1.614899↓	NO	NO	NO	NO
mmu04350↓	TGF-β signaling pathway	1.310092↓	NO	NO	NO	NO

#### 3.1.4 Verifying the DE genes after MTB infection through GEO database

In this study, significantly upregulated or downregulated DE genes in the TB model group vs. the normal group may also be confirmed in the GSE54992, GSE48027, GSE98461, GSE83456, GSE62525, GSE34608, GSE14361 datasets (Table 7). For example, significantly upregulated DE genes (*camp*, *cxcl9*, *cfb*, *gm4951, c4b, gm4841, ngp, serpina3g, and iigp1b*) after MTB infection in this study were also significantly upregulated in multiple databases, and significantly downregulated DE genes (*sult1d1* and *hhatl*) after MTB infection were also downregulated in the GSE14316 database. The gene *mcrs1* was significantly downregulated in both this study and GSE34608, but upregulated in GSE48027.

**TABLE 7:** Validation of DE genes by GEO database.

DE gene symbol	Fold change value of DE gene							
	TB model vs. normal control	GSE54992	GSE48027	GSE98461	GSE83456	GSE62525	GSE34608	GSE14361
*Camp*	35↑	1.58072↑		3.67021↑	0.94028↑		2.7781↑	
*Cxcl9*	46↑		0.3392↑				0.5998↑	5.9305↑
*Cfb*	30↑		3.5946↑		0.95932↑	0.7370↑		3.5183↑
*Gm4951*	21↑		4.2915↑					
*C4b*	26↑		2.3780↑					
*Gm4841*	24↑		6.2404↑					
*Ngp*	22↑		0.7865↑					
*Mcrs1*	9↓		1.1604↑				0.6370↓	
*Serpina3g*	23↑							2.7568↑
*Iigp1b*	20↑							0.5471↑
*Sult1d1*	9↓							2.4197↓
*Hhatl*	8↓							0.5090↓

#### 3.1.5 Verifying the DE genes after MTB infection through RT-qPCR

**FIGURE 3:**
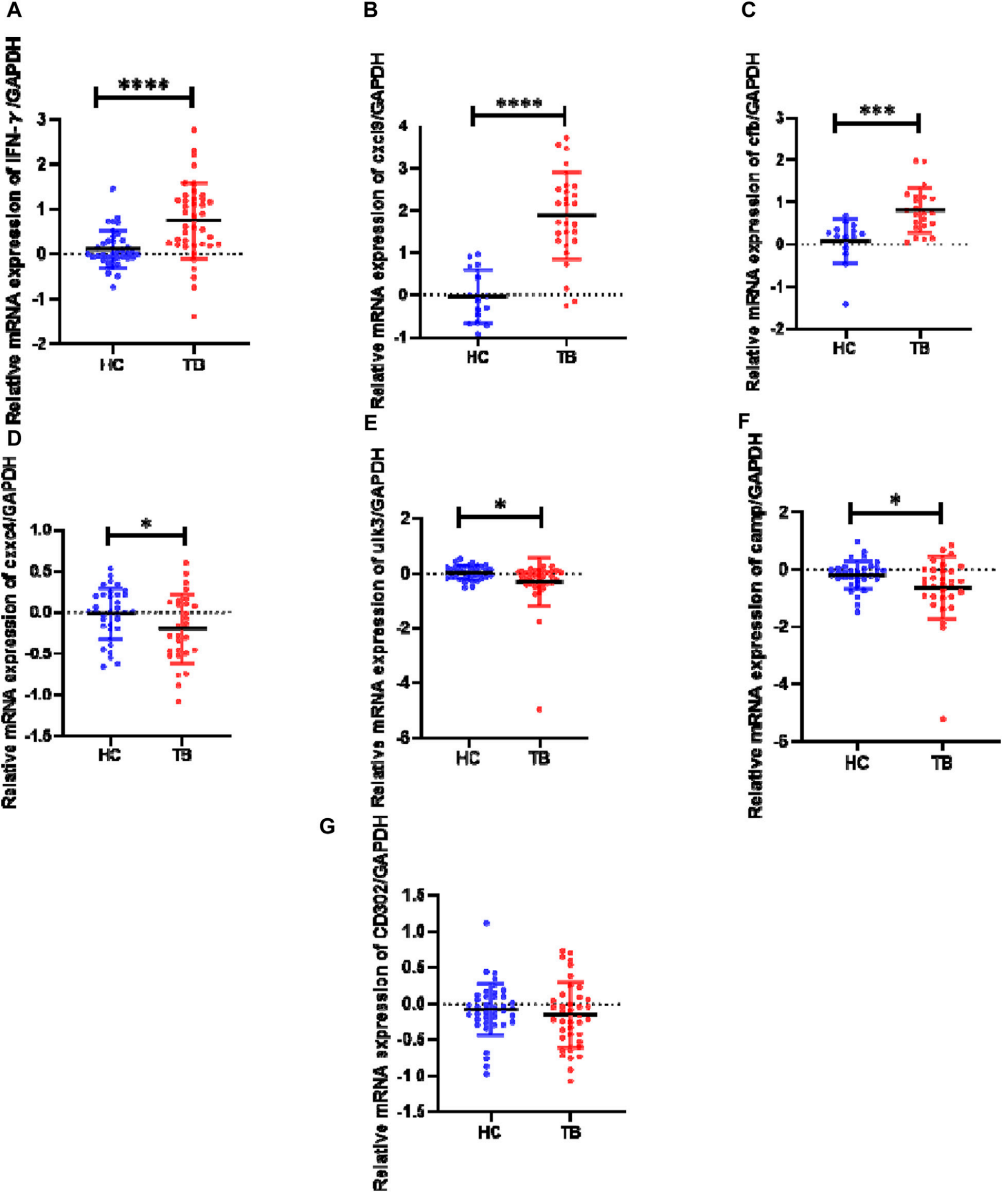
Expression levels of genes *ifn-γ*, *cxcl9*, *cfb*, *cxxc4*, *ulk3*, *camp*, and *cd302* in the human PBMC samples as determined by RT-qPCR HC (healthy controls): negative IGRA and without abnormality in lung CT; TB (tuberculosis): initial treatment TB patients with positive IGRA or smear or Xpert. The data between the two groups were tested by Student’s t-test. Results of cytokine relative expression were expressed as a scatter chart, in which **(A)** means *ifn-γ* (*p-value* < 0.0001); **(B)** means *cxcl9* (*p-value* < 0.0001); **(C)** means *cfb* (*p-value* = 0.0003); **(D)** means *cxxc4* (*p-value* = 0.0493); **(E)** means *ulk3* (*p-value* = 0.0227); **(F)** means *camp* (*p-value* = 0.0362); **(G)** means *cd302* (*p-value* = 0.4306).

The relative transcript expression levels of six genes (*cxcl9, camp, cfb, cxxc4, ulk3*, and *cd302*) in the TB model group vs. the normal group, were analyzed in the TB patients and healthy individuals by RT-qPCR to verify the reliability of profile results and *ifn-γ* was used as a positive control (Figure 3). The results showed that: In initially treated TB patients, the expressions of *ifn-γ, cxcl9,* and *cfb* were significantly higher than those of HCs (*p* < 0.0001, *p* < 0.0001, *p* < 0.001), and the expressions of *camp, cxxc4*, and *ulk3* were significantly lower than those of HCs (*p* < 0.05). However, there was no significant difference in the expression of *cd302* was no significance from that of compared to HCs (*p* > 0.05). The results of *cxcl9, cfb, cxxc4*, and *ulk3* verification were consistent with the results of gene expression profiles, while the results of *camp* verification were contrary to the results of expression profiles, and the results of *cd302* verification showed no significance.

### 3.2 Immune responses in mice after NBXH treatment

#### 3.2.1 DE genes in various dose NBXH groups

**FIGURE 4:**
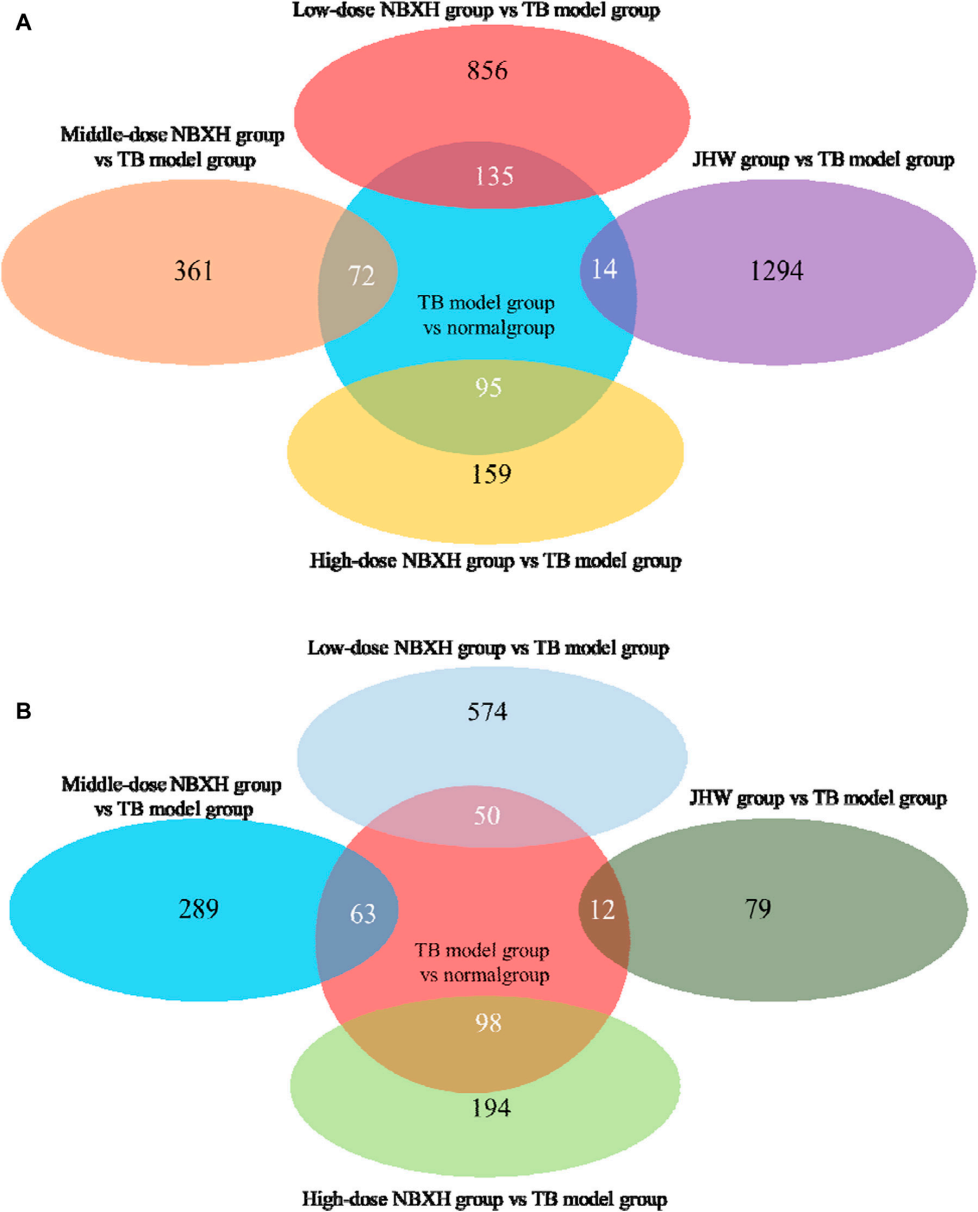
The DE gene Venn diagram between TB model group vs. normal group, JHW group vs. TB model group, and each dose NBXH group vs. TB model group. **(A)** Downregulated DE genes in the TB model group vs. normal group and upregulated DE genes in JHW group vs. TB model group and each NBXH group vs. TB model group; **(B)** Upregulated DE genes in the TB model group vs. normal group and downregulated DE genes in JHW group vs. TB model group and each NBXH group vs. TB model group, intersecting parts mean overlapping DE genes.

After treatment with low, middle, and high doses of NBXH, mice infected with MTB showed significant changes in gene expression *in vivo* (Table 2), and we found a negative correlation between the therapeutic dose of NBXH and the number of DE genes. Figure 4 reveals the number of overlapped DE genes between each dose of the NBXH group vs. TB model group and TB model group vs. normal group. The results showed that there were 50, 63, and 98 DE genes upregulated in the TB model group vs. normal group but downregulated in the low, middle, and high-dose NBXH groups vs. TB model group, respectively; there were 135, 72, and 95 DE genes downregulated in TB model group vs. normal group but upregulated in the low, middle, and high-dose NBXH groups vs. TB model group, respectively.

In addition, the top 10 upregulated and downregulated DE genes in the TB model group vs. normal group and their changes in low, middle, and high-dose NBXH groups vs. TB model group (Tables 3, 4) showed that: 1) The upregulated DE genes in the TB model group vs. normal group became downregulated in high-dose NBXH group vs. TB model group but had no significant difference in low-dose NBXH group vs. TB model group, middle-dose NBXH group vs. TB model group, and JHW group vs. TB model group. 2) The downregulated DE genes in the TB model group vs. normal group had no significant difference in the low-dose NBXH group vs. TB model group and the JHW group vs. TB model group, while most of the downregulated DE genes were significantly upregulated in the middle-dose NBXH group vs. TB model group and high-dose NBXH group vs. TB model group.

Therefore, we focused on the DE gene changes after the treatment with high-dose NBXH. The top 10 DE genes in the high-dose NBXH group vs. TB model group showed (Supplementary Tables S1, S2): 1) Of the top 10 upregulated DE genes in the high-dose NBXH group vs. TB model group, there also were 1, 5, and six upregulated DE genes in the JHW group vs. TB model group, low-dose NBXH group vs. TB model group, middle-dose NBXH group vs. TB model group, respectively. However, 9 DE genes were downregulated in the TB model group vs. the normal group, mainly related to information transmission in the nervous system and regulation of immune system function. 2) Of the top 10 downregulated DE genes in the high-dose NBXH group vs. TB model group, there also were 1, 4, and eight downregulated DE genes in the JHW group vs. TB model group, low-dose NBXH group vs. TB model group, and middle-dose NBXH group vs. TB model group, respectively. However, 6 DE genes were upregulated in the TB model group vs. the normal group, mainly related to nerve and endocrine regulation.

Although the low-dose NBXH group vs. TB model group had the most DE genes, there was no significant recovery effect on gene expression disorders caused by MTB infection. Middle-dose NBXH had a recovery effect on gene expression abnormalities caused by MTB infection, but the number or the FC values of recovered DE genes were not as well as those of high-dose NBXH. Although the high-dose NBXH group had the least number of DE genes, it had a significant corrective effect on most abnormal gene expression and pathways caused by MTB infection.

#### 3.2.2 GO analysis in various dose NBXH groups

The top 10 GO items in low-, middle-high-dose NBXH groups vs. TB model group, and JHW group vs. TB model group (Supplementary Figure S2) showed that: 1) Of the top 10 downregulated GO items in the low-dose, middle-dose, and high-dose NBXH group vs. TB model group, and JHW group vs. TB model group, there were 0, 4, 4, and four upregulated GO items in the TB model group vs. normal group, respectively. Of the top 10 upregulated GO items in the low-dose, middle-dose, and high-dose NBXH groups vs. TB model group, and JHW group vs. TB model group, there were 6, 2, 10, and 6 downregulated GO items in the TB model group vs. normal group, respectively. 2) In the high-dose NBXH group vs. TB model group, the upregulated CC items were mainly related to various membrane components, extracellular regions, and cell surfaces, such as membrane, cell surface, side of membrane, intrinsic component of membrane, and integral component of membrane; while the downregulated CC items were mainly associated with organelles and MHC molecules, such as organelle, MHC protein complex, extracellular region, MHC class I protein complex, MHC class I peptide loading complex, and intracellular organelle. 3) In the high-dose NBXH group vs. TB model group, the upregulated BP items were mainly related to the negative regulatory process and immunity, such as negative regulation of cellular process, cell adhesion, biological adhesion, negative regulation of biomineral tissue development, negative regulation of biomineralization, negative regulation of biological process, natural killer cell-mediated immunity, and regulation of epidermal growth factor-activated receptor activity; while the downregulated BP items involved various processes of occurrence and development, antigen processing and presentation, etc., such as cell differentiation, cellular developmental process, developmental process, anatomical structure development, TAP-independent antigen processing and presentation of endogenous peptide antigen *via* MHC class I *via* ER pathway, positive regulation of biological process; canonical Wnt signaling pathway. 4) In the high-dose NBXH group vs. TB model group, the upregulated MF items mainly involved various substance binding and enzyme activities, such as binding, MHC protein complex binding, phosphatidylinositol kinase activity, filamin binding, transferase activity (GTP-Rho binding), and MAP kinase activity; while the downregulated MF items mainly involved various substances binding, such as molecular function regulator, peptide antigen binding, CD8 receptor binding, β-2-microglobulin binding, TAP binding, binding, β-catenin binding, serine-type endopeptidase inhibitor activity, intracellular calcium activated chloride channel activity, and γ-catenin binding.

#### 3.2.3 Pathway enrichment analysis in various dose NBXH groups

**FIGURE 5:**
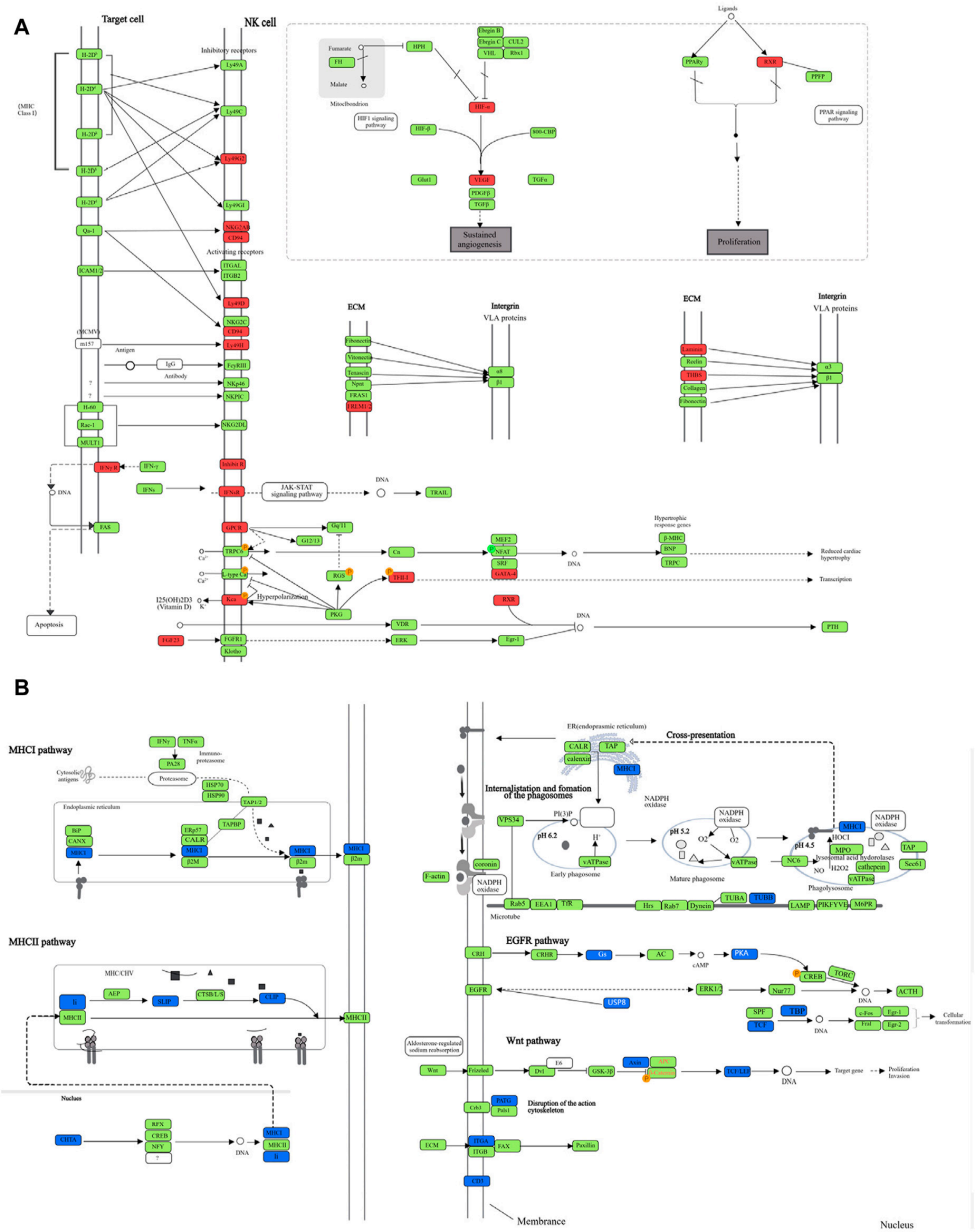
The DE genes in significantly upregulated and downregulated pathways after NBXH high dose group vs. TB model group. **(A)** The diagram of each pathway shown in Supplementary Table S3, and the significantly upregulated DE genes in each pathway after the high-dose NBXH treatment on mouse TB model; **(B)** The diagram of each pathway shown in Supplementary Table S4, and the significantly downregulated DE genes in each pathway after the high-dose NBXH treatment on mouse TB model. Red represents the upregulated DE genes, green represents unchanged genes, and blue represents downregulated DE genes.

The eight upregulated pathways in the high-dose NBXH group vs. TB model group and their changes in other dose NBXH groups vs. TB model group, TB model group vs. normal group, and JHW group vs. TB model group were shown in Supplementary Table S3, most of them were related to host immune response and endocrine such as natural killer cell-mediated cytotoxicity, parathyroid hormone synthesis, secretion and action. After summarizing the DE genes involved in eight pathways (Figure 5A), we found that although these pathways were involved in various functions, the changed genes in each pathway were certain, mainly related to cytokines and NK cell receptors such as CD94 and IFNsR. The top 10 downregulated pathways in the high-dose NBXH group vs. TB model group were shown in Supplementary Table S4, which were also related to host immunity and endocrine, such as antigen processing and presentation and phagosome. After summarizing the DE genes involved in the 10 pathways (Figure 5B), we found that downregulated DE genes were mainly observed in MHC molecular-related pathways.

#### 3.2.4 Verifying treatment-related DE genes through GEO database

Some significantly upregulated or downregulated DE genes in the high-dose NBXH group vs. TB model group were also confirmed in the GSE54992, GSE62147, and GSE48027 datasets (Table 8). For example, DE genes (*cd302*, *gm4841*, *ina*, and *cdh2*) significantly upregulated after MTB infection were significantly downregulated after high-dose NBXH treatment and chemotherapy in multiple datasets. Among some DE genes significantly downregulated after MTB infection, *lrrc74b* and *ulk3* were significantly upregulated after high-dose NBXH treatment and chemotherapy in both datasets, while *nrxn3* was significantly upregulated after high-dose NBXH treatment, but still showed downregulation after chemotherapy in the GSE54992 and GSE62147 datasets.

**TABLE 8:** Validation of the DE genes in the high-dose NBXH group vs. TB model group by GEO databases.

Gene symbol	DE gene Fold change value					
	Treatment time	12weeks	26 weeks			
	TB model group vs. normal group	NBXH high dose group vs. TB model group	GSE54992	GSE62147	GSE48027	GSE31348
*Lrrc74b*	11.29575↓	14.461↑			1.157475↑	
*Cd302*	14.70546↑	4.855↓			0.752155↓	
*Gm4841*	24.44741↑	3.846↓			1.86787↓	
*Nrxn3*	3.07623↓	14.984↑	1.93106↓	0.56505↓		
*Ulk3*	4.09779↓	11.672↑		0.68471↑		0.941↑
*Ina*	no	4.429↓		0.55435↓		
*Cdh2*	2.30694↑	3.917↓				1.49↓

#### 3.2.5 Verifying treatment-related DE genes through RT-qPCR

**FIGURE 6:**
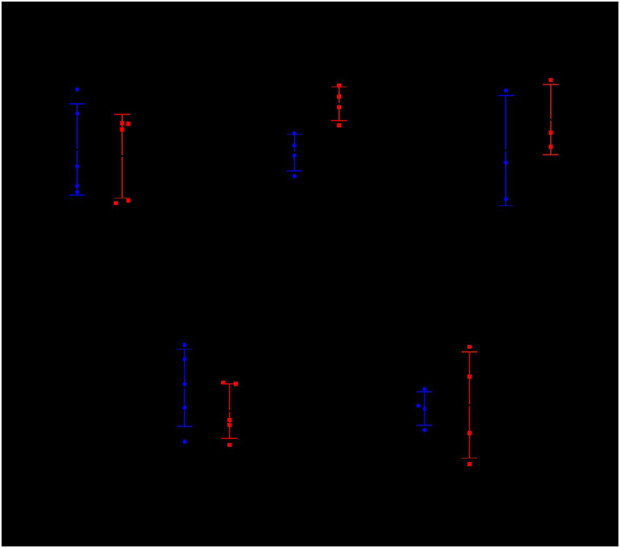
Transcript levels of *ifn-γ*, *cxcl9, cfb, cxxc4*, and *ulk3* genes in the human PBMCs samples as determined by RT-qPCR.

Using *ifn-γ* as the positive control group, DE gene *cxcl9, cfb, cxxc4*, and *ulk3* were further verified by RT-qPCR before and after chemotherapy combined with NBXH treatment in TB patients (Figure 6). The results showed that: the expressions of *ifn-γ* and *ulk3* showed no significant changes before and after treatment (*p* = 0.8381, *p* = 0.8888), while the expression of *cxcl9* was significantly upregulated (*p-value* = 0.0188), and the expression of *cfb* also showed an upregulated trend but without significant difference (*p-value* = 0.5534), and the expression of *cxxc4* was downregulated but also without significant difference (*p-value* = 0.2022).

TB patients before treatment (BT): initially treated pulmonary TB patients with positive IGRA or smear or Xpert; TB patients after treatment (AT): initially treated pulmonary TB patients with positive IGRA or smear or Xpert after treatment for ≥2 months.

The data between the two groups were tested by a Student’s t-test. The results were expressed as a scatter chart, where A means *ifn-γ* relative expression (*p-value* = 0.8381); B means *cxcl9* relative expression (*p-value* = 0.0188); C means *cfb* relative expression (*p-value* = 0.5534); D means *cxxc4* relative expression (*p-value* = 0.2022); E means *ulk3* relative expression (*p-value* = 0.8888).

## 4 Discussion

TCM combined with chemotherapeutics in the treatment of TB has achieved significant clinical efficacy. For example, JHW, a Chinese medicine preparation, has been applied to clinics with a positive anti-TB therapeutic effect. Our previous studies have proved that NBXH has significant effects on the treatment of TB and alleviates the side effects of chemotherapy (Liang et al., 2017; Liang et al., 2021). This study attempts to explain the mechanism of the host responses against MTB infection in mice and the mechanisms of TCM preparation NBXH in the early treatment of acute MTB-infected mouse model through the gene expression microarray technology, to provide scientific explanation and verification for TCM.

### 4.1 Activation of anti-TB immunity in mice after MTB infection

After the mice were infected with MTB 3 months, the top 10 significantly upregulated DE genes in the TB model group vs. normal group were mainly related to anti-TB immune responses (Table 2). *Cxcl9*, encoding the monokine induced by interferon-γ (MIG), was the most significantly upregulated DE gene. CXCL9 binds to its receptor CXCR3 to recruit CXCR3^+^ cells, such as monocytes, CD4^+^ Th1 cells, and CD8^+^ cytotoxic T Cells, inducing Th1 immunity and inhibiting Th2 immunity (De Benedetti et al., 2021). It participates in immune regulation and inflammatory processes and plays an important role in host anti-TB immunity. Several studies have shown that *cxcl9* gene expression and serum levels were significantly upregulated in hosts after MTB infection (Fan et al., 2022; Khalid et al., 2023; Li et al., 2023; Sampath et al., 2023). In this study, both the results of the GEO database and RT-qPCR validation also showed that the expression of the *cxcl9* gene was significantly upregulated after MTB infection, and *cxcl9* can serve as a potential diagnostic marker of MTB infection. Other studies have shown that *cxcl9* gene expression and serum levels were significantly downregulated after effective treatment, which can be used as potential therapeutic monitoring biomarkers (Ambreen et al., 2021; Khalid et al., 2023). The *cxcl9* expression in our result was consistent with other study, which means the treatment was effective. However, the opposite result in RT-qPCR assay may indicate that the patient’s condition has not been fully controlled and further treatment is needed. Cationic antibacterial peptide (CAMP), a member of the antimicrobial peptide family (AMP), is mainly expressed in epithelial cells (such as intestine, airway, skin, etc.,) and innate immune cells (such as neutrophils, natural killer cells, mast cells, dendritic cells, monocytes, and macrophages). As an endogenous effector molecule in innate immunity, CAMP can directly inhibit pathogen activity and regulate host innate and adaptive immune responses, including inducing pro-inflammatory effects, promoting autophagy, etc., (Bucki et al., 2010; Chen et al., 2017; Bei et al., 2019). Therefore, CAMP has broad application prospects in anti-TB treatment due to its broad-spectrum antibacterial activity (Dlozi et al., 2022). Our results and the results of 3 GEO datasets indicated that CAMP plays an important role in resisting MTB invasion. However, the opposite result in RT-qPCR assay may be related to the suppression of immune system function in TB patients. In addition to *cxcl9* and *camp*, the significantly upregulated DE genes in the TB model group vs. normal group also included *cfb* (Shi et al., 2022), *c4b* (Daher et al., 2015; Wang et al., 2023), and *ngp* (Kathamuthu et al., 2021), which are closely related to the host’s anti-TB immune function. The complement system is an important component of innate immunity. Complement factor B (CFB) and complement C4b are members of the complement family, playing important roles in mobilizing both innate and adaptive immunity against pathogen invasion (Conigliaro et al., 2019). CFB is activated through the alternative complement pathway (AP) (Matsumoto et al., 1997), then activates the NF-κb inflammatory pathway to secrete inflammatory cytokines and recruit macrophages (Wang et al., 2009). C4b is activated through the classical complement pathway (CP) and lectin pathway (LP). The C1 complex cleaves C4 to produce C4a and C4b, and C4b further activates C3, initiating a series of complement cascade reactions, and forming complement complexes to exert immune function (Werner and Criss, 2023). Our results of the animal experiments, GEO datasets, and RT-qPCR validation all proved that the expressions of *cfb* and *c4b* were significantly upregulated after MTB infection, indicating that the host activates innate immune responses and participates in inflammation responses to clear invasive pathogens through the alternative pathway and the classical activation pathway, respectively (Utaiwat et al., 2021). Neutrophilic granule protein (NGP), a member of the cystatin superfamily, is mainly expressed in granulocytes (Moscinski and Hill, 1995). NGP can enhance phagocytosis of macrophages, act as an antagonist of lipopolysaccharide (LPS), activate TLR4-NF-κb signaling pathway to induce IL-10 secretion, inhibit TNF-α and IL-1β secretion (Liu et al., 2020), enhance the host’s immune defense ability, and inhibit inflammatory responses. The GEO validation results also showed that *ngp* was highly expressed in TB patients to participate in inflammatory responses. *Serpina3g*, a member of the serine protease inhibitor superfamily, participates in various immune activities. As a target of the JAG-STAT signaling pathway, SERPINA3G plays an important role in inducing GM-CSF synthesis and regulating the function of neutrophils and B lymphocytes, promoting anti-inflammatory, antioxidant, and anti-fibrotic effects (Li et al., 2014). Meanwhile, the expression level of *serpina3g* has also been reported to be positively correlated with the degree of pulmonary injury induced by chronic obstructive pulmonary disease (COPD) (Uysal and Uzun, 2019). GEO validation results also showed that *serpina3g* was highly expressed in TB patients, which proved that this gene played an important role in the inflammatory response of TB.

After MTB infection in mice for 3 months, the top 10 significantly downregulated DE genes mainly involved stress responses, cellular processes, and immune responses, etc., (Table 3). Among them, SULT1D1, as a sulfonyltransferase, is mainly expressed in the kidneys and livers of mice. Its main biological function is to regulate the sulfonation metabolism of catecholamines, playing an important role in the inactivation of endogenous dopamine derived compounds (including catecholamines) (Tsoi et al., 2001; Wong et al., 2010). GEO verification results showed that the expression of *sult1d1* was downregulated in TB patients, which may reduce the inactivation of catecholamines, thus maintaining the stress response ability of TB patients. CXXC finger protein 4 (CXXC4) is the negative regulatory factor of Wnt/β-catenin signaling pathway and is also able to suppress the activation of NF-κB and p38 mitogen activated protein kinase (MAPK) pathway (Han et al., 2017; Li et al., 2020c), which are involved in the chronic inflammatory responses of TB (Holscher et al., 2020; Poladian et al., 2023). It was clearly confirmed through RT-qPCR validation. CXXC4 deficiency was the host’s desire to activate Wnt/β-Catenin signaling pathway to enhance the activities of the NF-κB and MAPK pathways, which promotes macrophage activation, phagosome maturation, and the release of various cytokines to control MTB growth (Holscher et al., 2020; Poladian et al., 2023). *Grip2* is a key regulatory factor in the development of innate CD8^+^ T Cells (i8Ts) (Jo et al., 2020), which is nonconventional αβT cells that exhibit features of memory phenotype. Downregulation of *grip2* expression by MTB infection will affect the differentiation and maturation of CD8^+^ T Cells.

The results of GO analysis and pathway analysis also confirmed that the NK cell surface receptors, Wnt signal pathway, and TGF-β signal pathway were downregulated in mice after MTB infection, which weakens multiple processes such as adhesion, differentiation, endocrine, and transportation, and also promoted the host’s recognition, processing, and presentation of antigens, as well as immune defense responses. The body was also striving to mobilize the immune system to resist damage caused by MTB invasion. The impact of MTB infection on host gene expression is comprehensive, even disrupting gene expression in nervous and endocrine systems. The neurohumoral regulatory system extensively regulates immune system function. In recent years, more and more researchers have paid attention to the regulatory role of the neurohumoral system on the immune response caused by exogenous pathogen infections (Huang et al., 2019; Malin et al., 2020; Kim et al., 2022). For example, activation of the cholinergic anti-inflammatory pathway (CAP) can limit lung infection (Su et al., 2010), and reduce inflammatory damage, as well as inhibit the NF-κB pathway in splenic macrophages to reduce inflammatory responses while inhibiting the migration of splenic macrophages (Borovikova et al., 2000; Wang et al., 2003). In addition to the vagus nerve, the sympathetic and parasympathetic nerves can also regulate inflammatory responses. Gang Cao et al.’s research showed that neuron-derived neuropeptide Y (NPY) can precisely regulate the inflammatory responses of the spleen, acting as the "interaction language” between the immune system and central nervous system (Yu et al., 2022). Therefore, the DE genes of the nervous and endocrine systems that exhibit significant changes after MTB infection may also be involved in the neuroimmune regulation process, and we will further investigate and clarify it.

### 4.2 Relationship between NBXH dose and effect

The dose-effect relationship results showed: 1) The dosage change of NBXH influenced the therapeutic effect, and within a certain dosage range, the therapeutic effect will have a positive correlation with the change of NBXH dosage. The dosage of TCM is an extremely important part of TCM prescriptions, which is closely related to clinical efficacy. Chen JQ et al. and Chen YY et al. used metabolomics technology to clarify the dose-effect relationship of TCM (Chen et al., 2020; Chen et al., 2022). Our study firstly revealed the dose-response relationship through transcriptomics, then combined with traditional pharmacological and toxicological studies can provide a basis for determining the clinical dosage of NBXH granules. 2) High-dose NBXH can enhance immune system function, reduce inflammatory damage, and play an anti-TB effect. JHW also had a partial recovery effect on the abnormal gene expression caused by MTB infection, but the recovery effect is not as good as high-dose NBXH. Moreover, the targets and regulatory pathways of the anti-TB effects of JHW and NBXH are different, indicating that these two TCM prescriptions exert anti-TB effects through different mechanisms of action.

### 4.3 Anti-TB mechanism of NBXH

The most significantly upregulated DE gene in high-dose NBXH group was *nrxn3*, which encodes a nerve cell adhesion molecule necessary for synaptic formation and maintenance, playing a role in the neuromuscular junction, synaptic formation, maturation, differentiation, and intercellular signaling processes. Therefore, high or low *nrxn3* expression is associated with a variety of neurological and psychiatric diseases (Kasem et al., 2018). The lack of NRXN3 can affect synaptic development, synaptic signaling, and neurotransmitter release, and the downregulation of *nrxn3* expression has been identified as the highest-risk gene associated with Alzheimer disease (AD) and senescence (Zheng et al., 2018). Meanwhile, *nrxn3* is also involved in the occurrence or metastasis of various tumors (Sun et al., 2013). There is currently no literature reporting its role in pathogen infection or host anti-infection. This study found for the first time that *nrxn3* expression was downregulated after MTB infection. The current study showed that TB patients had a higher risk of AD than non-TB patients. TB may be associated with an increased risk of AD, and inflammation may play a key role in AD. The GEO verification results showed that *nrxn3* expression in TB patients decreased after chemotherapy, but current studies have shown that rifampicin can have therapeutic effects on AD through anti-inflammatory, anti-tau, anti-amyloid, and cholinergic effects (Yulug et al., 2018). High-dose NBXH therapy significantly upregulates *nrxn3*, which may reduce the risk of AD and is worthy of further exploration. *Ulk3*, as a feature gene of autophagy, is a key target of the mTOR signaling pathway, and its expression can activate the cellular autophagy process (Gonzalez-Rodriguez et al., 2022), which is an important part of host anti-TB immunity (Lam et al., 2017). The results of GEO and RT-qPCR indicate that both chemotherapy and NBXH treatment can enhance the anti-TB autophagy effect of the body. In this study, it was found for the first time that *btbd17*, *krt72*, *otop3t*, and *gm10050* were significantly downregulated after MTB infection, while significantly upregulated after NBXH treatment. There were few reports on these genes, and no TB-related dataset was found in the GEO database, so further research is needed.

There are currently no reports in the field of TB on the top 10 significantly downregulated DE genes after high-dose NBXH treatment, and most genes can also not be retrieved from relevant datasets in the GEO database. Among them, *mageb16, cdkn2b*, and *cdh2* genes are associated with tumors. The *mageb16* gene, most significantly downregulated in this study, usually encodes tumor antigens and is also a DE gene or protein in many genomics research results such as gastric cancer (Zaragoza-Huesca et al., 2022), colorectal cancer (Juhari et al., 2021), bladder cancer (Liu et al., 2016), and other tumor diseases, as well as feline abdominal virus infection (Shuid et al., 2015), indicating that it may play an important role in tumorigenesis. *Cdkn2b* encodes the tumor suppressor gene *p15ink4b*, which is closely linked to the *cdkn2a* chromosome and participates in the cell cycle and senescence. *Cdkn2b* is often inactivated due to deletion, methylation, or mutation in various tumors, leading to tumor progression and being considered an inhibitor of tumors (Jang et al., 2019; Yang et al., 2021; Hjazi et al., 2023). *Cdh2*, a member of the Cadherin family, regulates many biological processes and has been proven to be closely associated with various cancers (Kourtidis et al., 2017). Cancer patients with high *cdh2* expression usually have poorer prognosis, while those with low *cdh2* expression have improved prognosis (Ma et al., 2016; Chen et al., 2018). This study found for the first time that MTB infection does not affect *mageb16* expression, but *cdkn2b* and *cdh2* expressions are both significantly upregulated. It was suggested that persistent tissue damage caused by long-term chronic inflammation of TB, cell proliferation induced by damage, and cicatrix formed by tissue repair were closely related to the occurrence of lung cancer. In addition to the damage recovery effect, further exploration is needed to determine whether NBXH also has a certain anti-cancer effect. Three genes related to anti-TB immunity, *serpinb6a, cd302*, and *gm4841*, were significantly upregulated after MTB infection. *Serpinb6a* can inhibit cathepsin G (CatG) activity in monocyte and neutrophil-mediated immune inflammatory responses, prevent cell apoptosis, promote cell survival, and regulate inflammatory responses (Burgener et al., 2019). Therefore, downregulation of *serpinb6a* expression after NBXH treatment can enhance macrophage inflammatory response and apoptosis, which is beneficial for clearing invading MTB and reducing the inflammatory damage caused by neutrophils. CD302, a protein containing a C-type lectin domain, is an important pattern recognition receptor (PRR) in the innate immune system. Its biological function is related to cell adhesion and migration, as well as phagocytosis and endocytosis, and it participates in the host’s resistance to the invasion and infection of various pathogenic microorganisms. It is mainly highly expressed in the liver, lungs, lymph nodes, spleen, and myeloid cells (such as monocytes, granulocytes, macrophages, and DC cells) (Kato et al., 2007; Lo et al., 2016; Peng et al., 2023). After NBXH treatment, the elevated *cd302* was downregulated by one-third, indicating that NBXH regulated the host’s anti-TB responses by inhibiting the phagocytic and bactericidal activity of monocytes/macrophages. In addition, *gprc5b*, which is widely expressed in the pancreas, is an important regulatory factor for pancreatic function, and its high expression leads to β cells being sensitive to the harmful effects of cytokines and increases the apoptosis induced by cytokines, which is related to type II diabetes. The downregulation of *cd302* can increase insulin secretion and promote β cell survival, which makes it an attractive target for developing new medicine to treat type II diabetes and its complications (such as diabetes nephropathy) (Soni et al., 2013; Atanes et al., 2018; Tao et al., 2022). NBXH treatment can downregulate *gprc5b* expression, which is beneficial to prevent TB with diabetes. There are also three genes (s*vs3a, vmn1r65*, and *ina*) related to molecular activity or cell structure. Currently, there are few research reports, and their role in anti-TB is not yet clear. GEO verification results only have confirmed that *ina* was significantly downregulated after treatment. Therefore, these genes as targets for NBXH treatment still need further exploration to explain their clinical significance.

The GO analysis and pathway analysis results showed that the antigen processing and presentation process was downregulated by a quarter compared to the TB model group vs. the normal group and NK cell-mediated cytotoxicity was significantly enhanced. The above analysis indicates that NBXH treatment can enhance anti-TB immunity such as autophagy, NK cell function, and apoptosis, which is beneficial for clearing intracellular MTB (Qin et al., 2023). At the same time, NBXH also had a certain inhibitory effect on immune regulatory targets that are overactivated during immune system activation.

In conclusion, MTB infection led to widespread changes in host gene expression and mainly stimulated the host’s anti-TB immune responses. The treatment using high-dose NBXH partially repaired the abnormal gene expression caused by MTB infection, further strengthened the anti-TB immunity in areas such as autophagy and NK cell-mediated cytotoxicity, and had a certain inhibitory effect on overactivated immune regulatory factors to alleviate immune damage. This study is the first to reveal the dose-response relationship of NBXH through transcriptomics, and further elucidate the mechanism of MTB infection and NBXH therapy, providing the scientific basis for TCM treatment of TB.

## Data Availability

The authors confirm that the data supporting the findings of this study are available within the article/Supplementary Material. The transcriptome data is available at the GEO database, accession number: GSE276937. Further enquiries can be directed to the corresponding authors.
