# Supplementary material for: Transcriptomics-based anti-tuberculous mechanism of traditional Chinese polyherbal preparation NiuBeiXiaoHe intermediates
Source: Front Pharmacol. 2024 Sep 19;15:1415951. doi: 10.3389/fphar.2024.1415951 (PMC11446850; doi:10.3389/fphar.2024.1415951)
Supplement: Supplementary file 1 [file DataSheet1.docx]

A B C


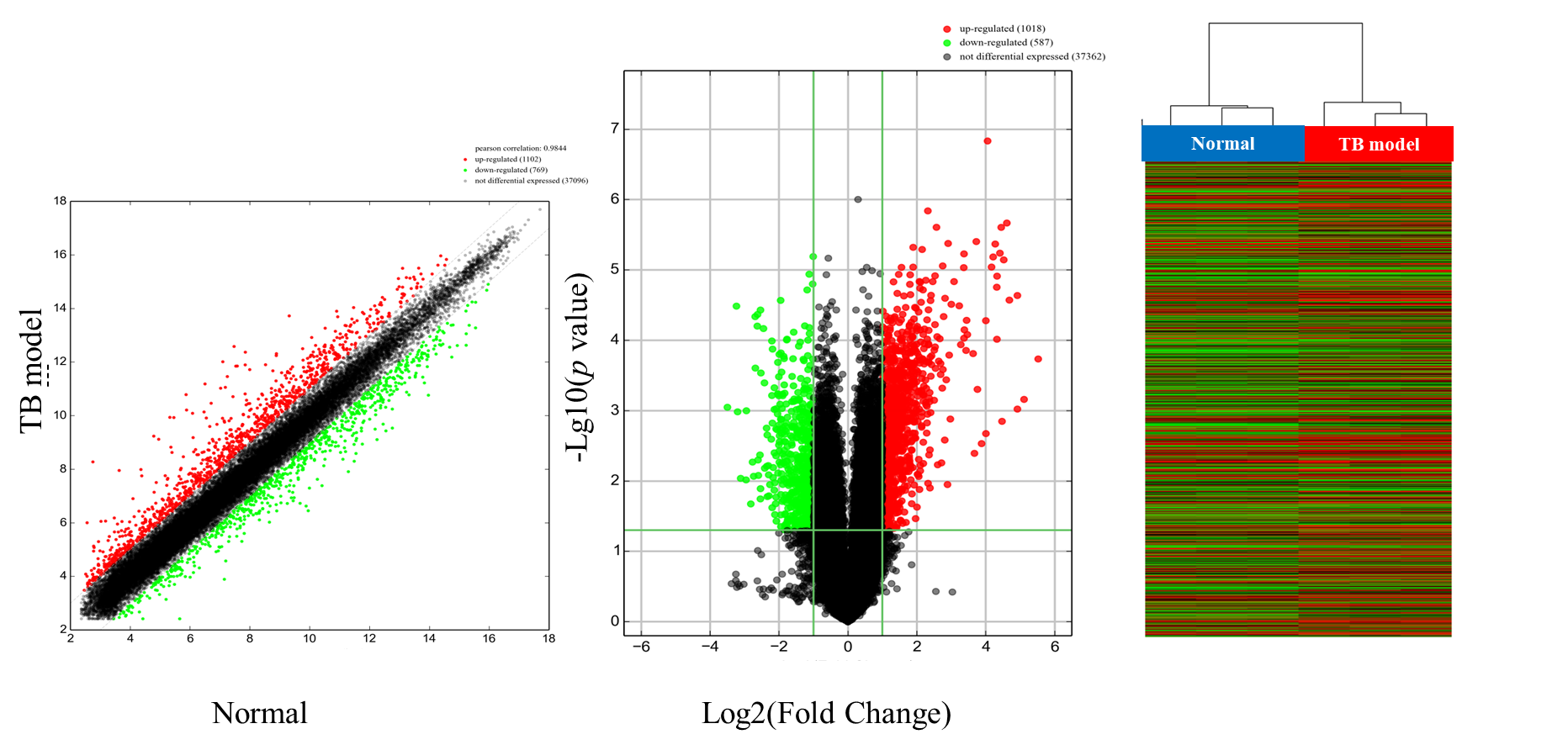


a


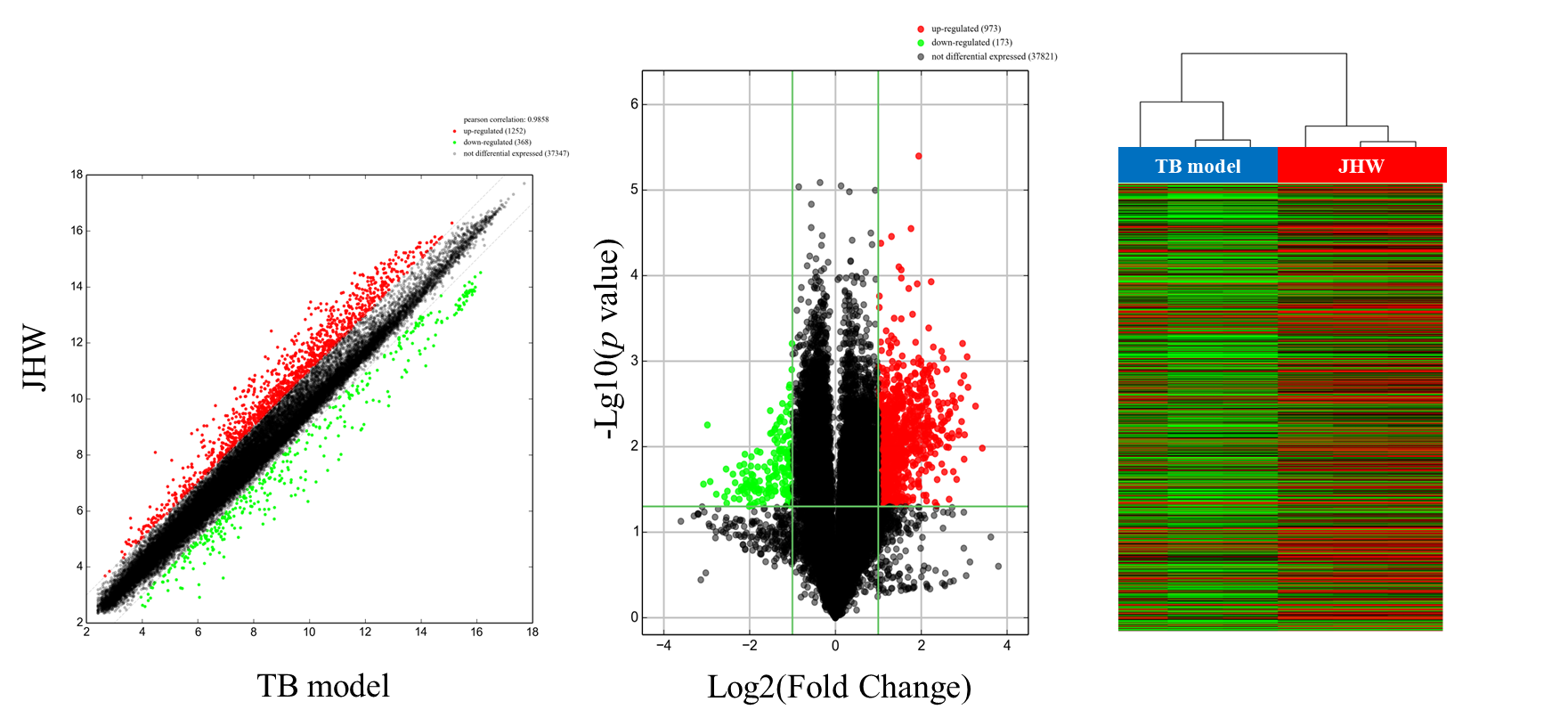


b


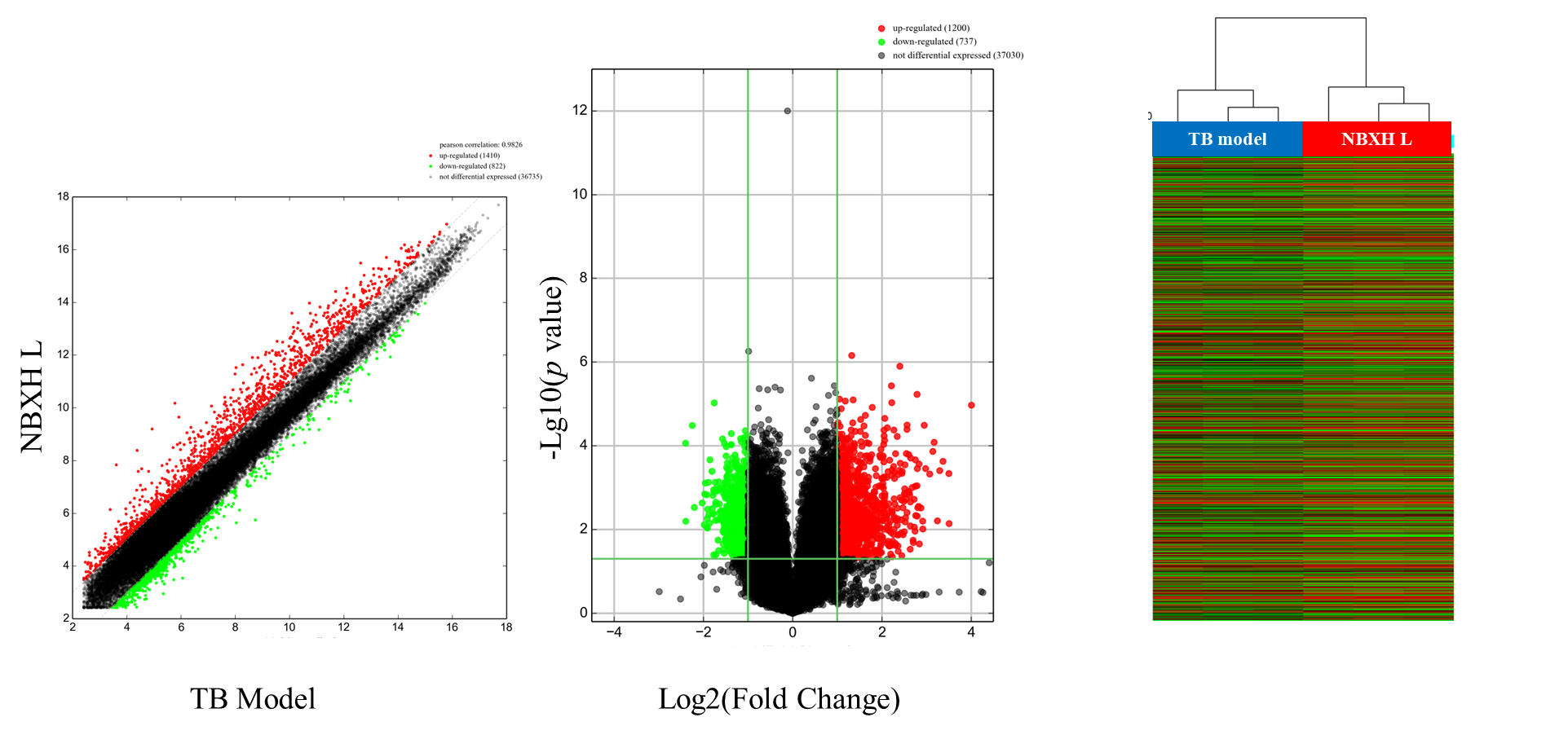


c


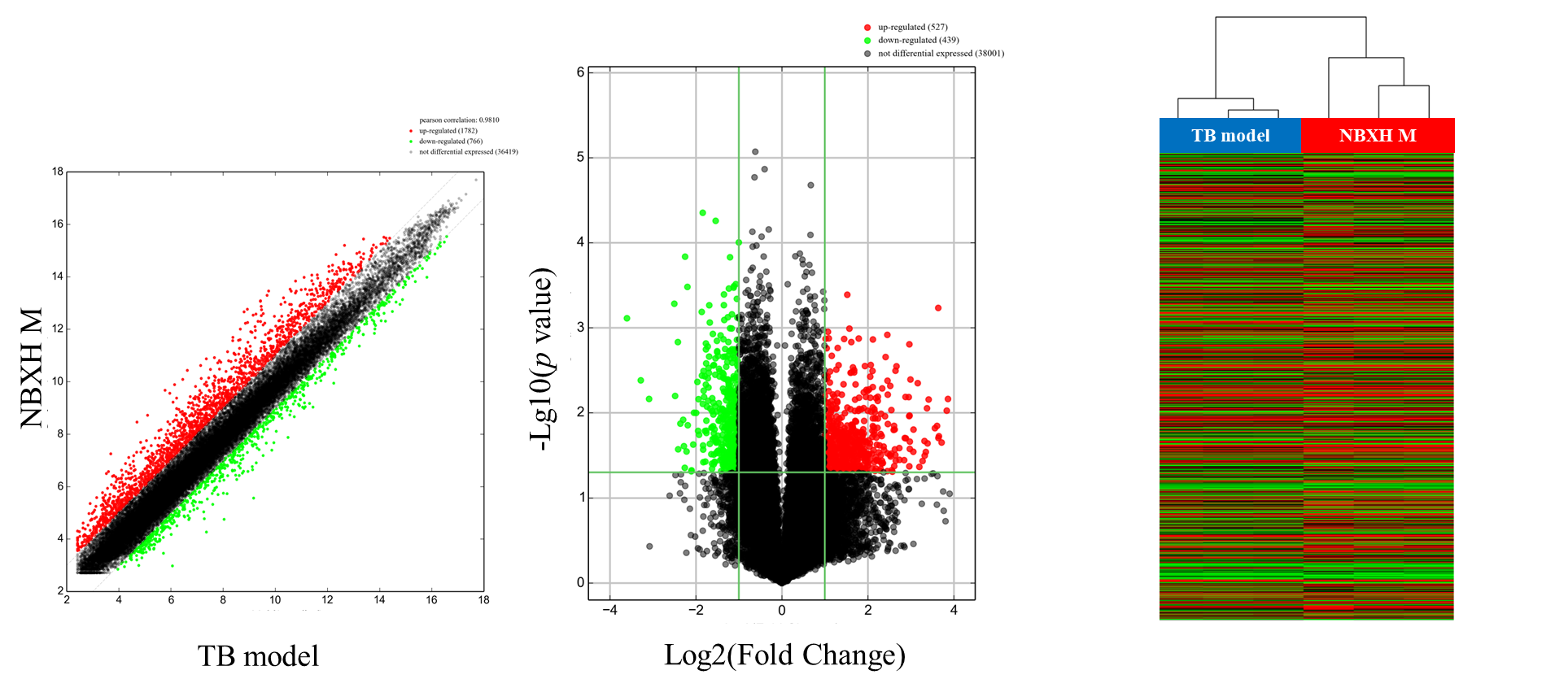


d


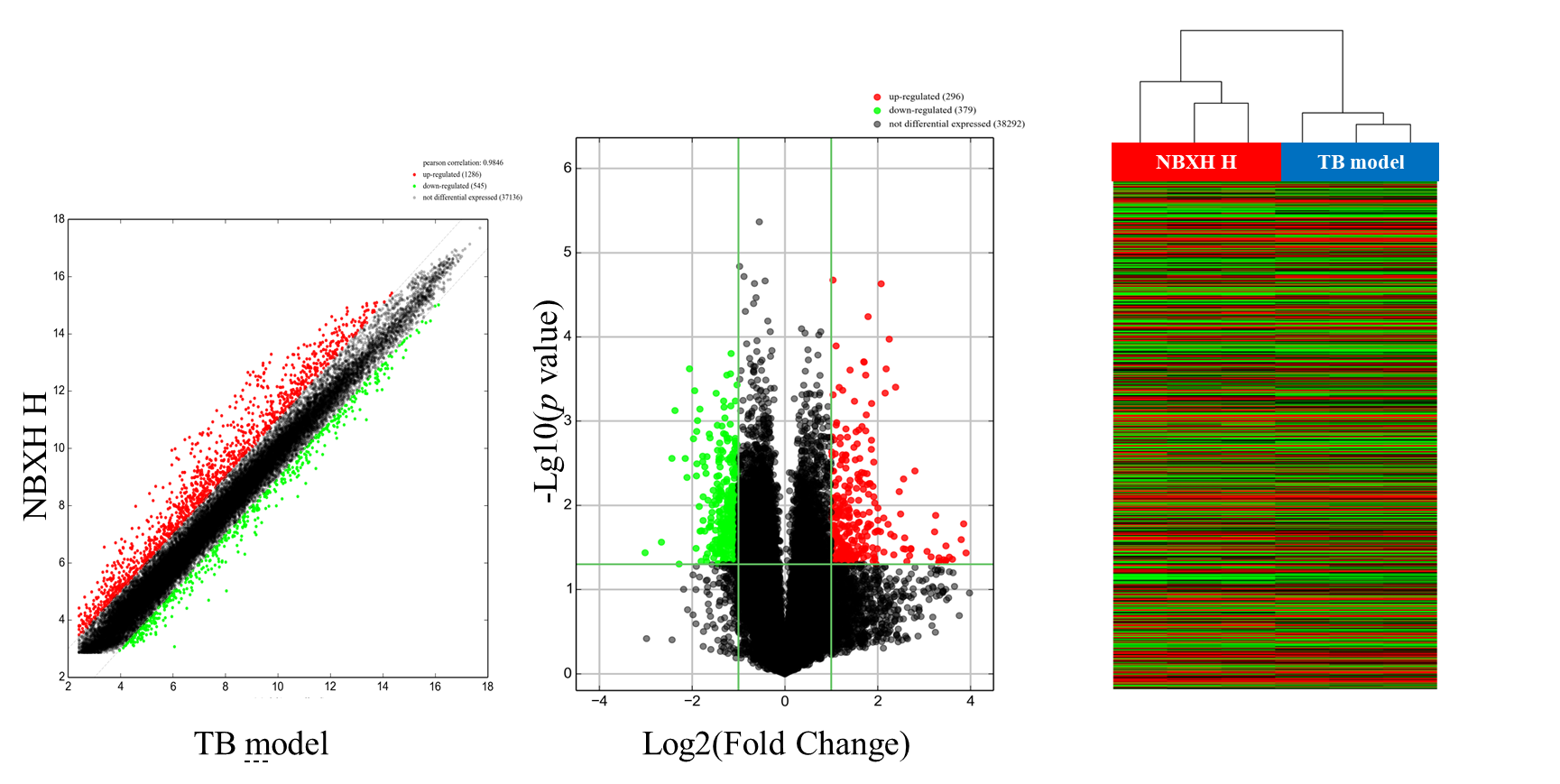


e

**Supplementary Figure 1 Scatter-plot, volcano plots, and hierarchical clustering visualized the gene expression variation between the TB model group and the normal group (a), or JHW treatment group and TB model group (b), or each NBXH treatment group and TB model group (c-e).**

(A) Scatter-plot, the values of the X and Y axes in the Scatter-plot are the averaged normalized signal values of the normal mouse group, mouse TB model group, JHW group, low-dose NBXH group, middle-dose NBXH group, and high-dose NBXH group (log2 scaled). The gray lines are Fold Change lines (the default fold change value given is 2.0). The genes above the top gray line and below the bottom gray line indicated more than a 2-fold change of gene expression between the two groups (n = 3). (B) Volcano plots, are constructed by using fold-change values and P-values. The vertical green lines correspond to 2.0 folds of up-regulated and down-regulated expression, respectively, and the horizontal green line represents a P-value of 0.05.

(C) Hierarchical clustering map, cluster analysis arranges samples into groups based on their expression levels. The dendrogram shows the comparison of gene expression differences between the two groups (3 samples). “Red” indicates the up-regulated expression of DE genes with statistical significance, and “green” indicates the down-regulated expression of DE genes with statistical significance.

**Up-regulated DE genes Down-regulated DE genes**

**TB model group vs. Normal group**


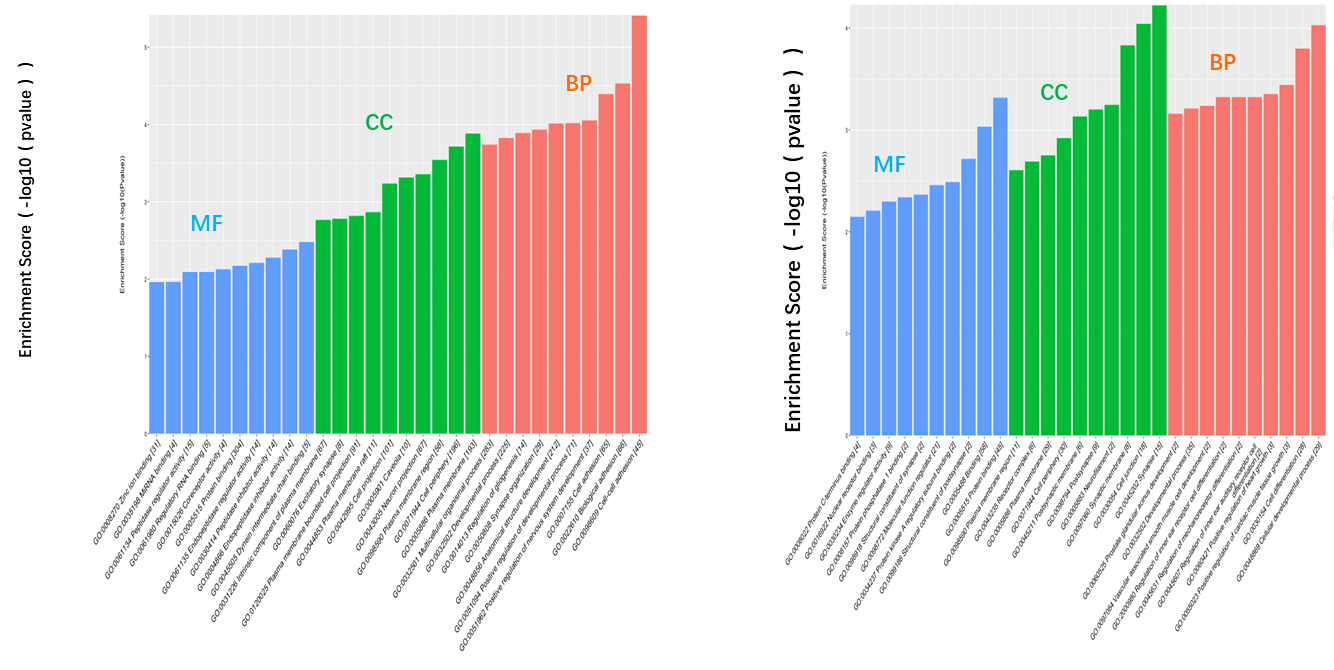


**JHW group vs. TB model group**


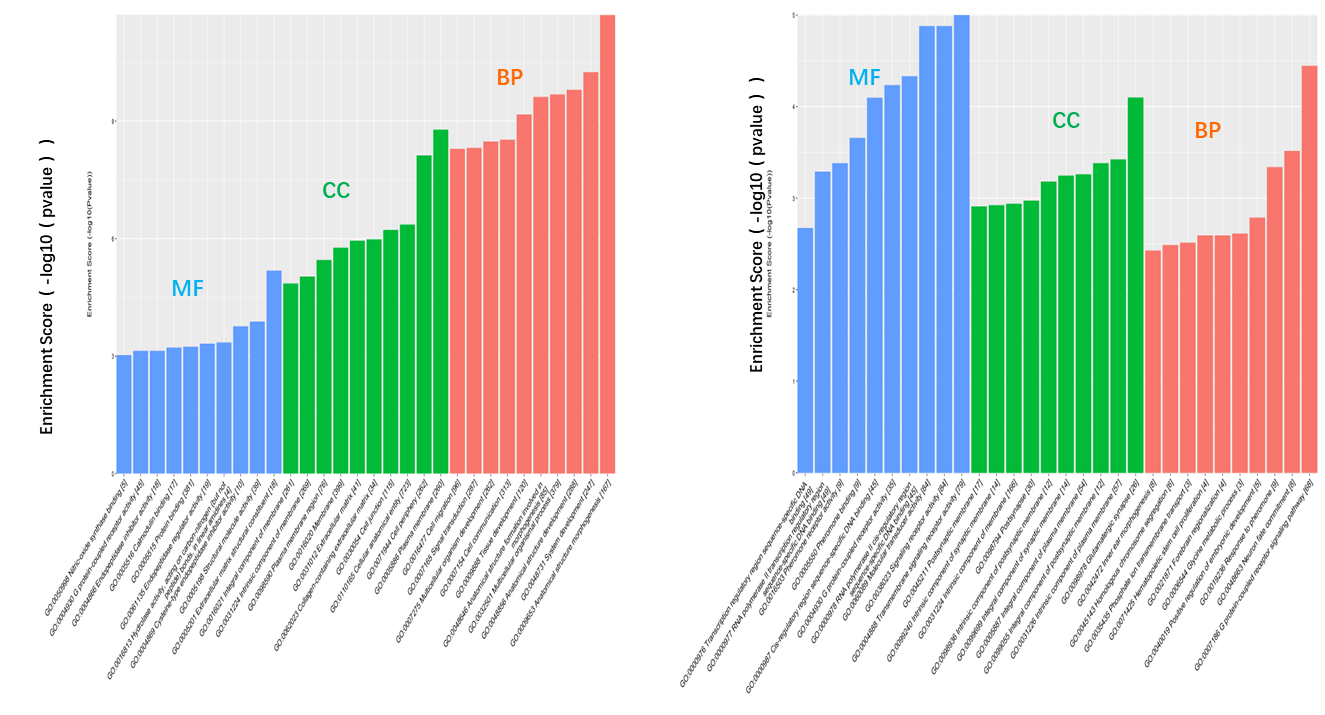


**Low-dose NBXH group vs. TB model group**


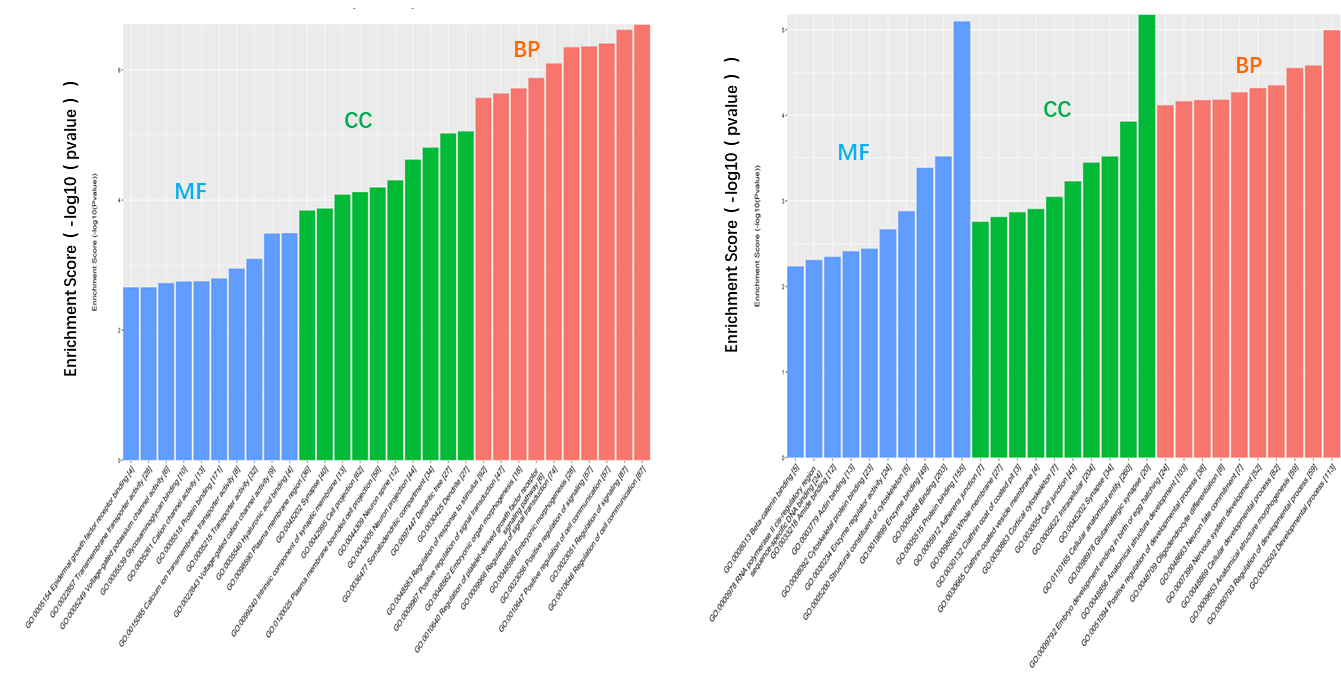


**Middle-dose NBXH group vs. TB model group**


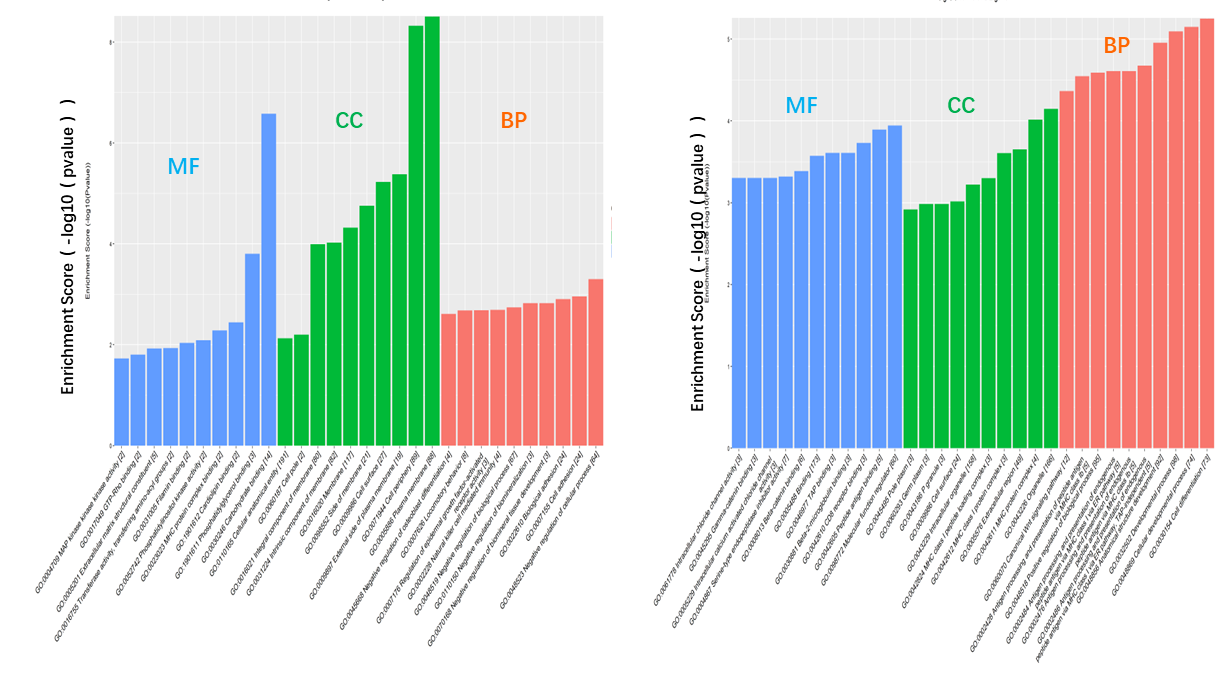


**High-dose NBXH group vs. TB model group**

**Supplement Figure 2. The top 10 GO analysis terms of significant DE genes in TB model group vs. normal group, JHW group vs. TB model group, and each NBXH group vs. TB model group.**

Significant DE genes in the TB model group vs. normal group, JHW group vs. TB model group, and each NBXH group vs. TB model group were respectively identified in biological process (BP), cellular component (CC), and molecular function (MF), respectively. The X-axis coordinate represents the top 10 terms of enrichment scores (sorted by P-value), and the Y-axis represents enrichment scores (scaled by p-value Log10). The P-value represents the enrichment significance of the GO item in the DE gene list, and the smaller the P-value is, the more significant the enrichment is (P≤0.05 is significant). Blue columns represent MF, green columns represent CC, and red columns represent BP.

Supplementary Table 1：The top 10 significantly upregulated DE genes in the high-dose NBXH group vs TB model group and their changes in other dose NBXH groups vs TB model group, JHW group vs TB model group, and TB model group vs normal group

| **GenBank accession** | **Gene symbol** | **NBXH groups vs TB model group** | | | | **JHW group vs TB model group** | **TB model group vs normal group** | **Annotation** |
| --- | --- | --- | --- | --- | --- | --- | --- | --- |
|  |  | **High dose** | | **Middle dose** | **Low dose** |  |  |  |
| NM_001198587 | Nrxn3 | 15↑ | no | | no | no | 3↓ | Neurexin 3, encodes a synaptic adhesion molecule of nerve cells and is involved in signal transmission between synapses of neurons. High or low nrxn3 expression is associated with a variety of neurological and psychiatric diseases[1], and is also involved in the occurrence or metastasis of a variety of tumors [2-4]. |
| NM_029053 | Lrrc74b | 14↑ | 12↑ | | 6↑ | no | 11↓ | Leucine-rich repeat-containing 74B, is a protein-coding gene. Its function is not yet clear (NCBI). |
| NM_198662 | 9430007A20Rik | 14↑ | 10↑ | | 3↑ | no | no | AADACL4 family member 1， has the activity of hydrolase and carboxylate hydrolase (NCBI). |
| AK082145 | C230013L11Rik | 12↑ | no | | no | 3↑ | 4↓ | - |
| NM_027895 | Ulk3 | 12↑ | 13↑ | | 7↑ | no | 4↓ | Unc-51 like kinase 3, is a serine/threonine protein kinase that acts as a regulator of Sonic Hedgehog (SHH) signaling and autophagy. |
| NM_026634 | A930018P22Rik | 11↑ | 15↑ | | 4↑ | no | 5↓ | - |
| NM_028055 | Btbd17 | 11↑ | 12↑ | | no | no | 5↓ | BTB domain containing 17, is predicted to be involved in negative regulation of viral genome replication and response to virus (NCBI). |
| NM_213728 | Krt72 | 11↑ | 9↑ | | no | no | 3↓ | Keratin 72, is an intermediate filament protein responsible for the structural integrity of epithelial cells. Its expression was positively correlated with CD4^+^ T cell infiltration in [prostate cancer](https://lib.plagh.cn/s/com/sciencedirect/www/G.https/topics/medicine-and-dentistry/prostate-cancer)[5]. |
| NM_001347647 | Otop3 | 11↑ | no | | no | no | 4↓ | Otopetrin 3, belongs to a newly identified family of proton (H+) channels activated by extracellular acidification, has proton channel activity, and is involved in proton transmembrane transport [6]. |
| AK047630 | Gm10050 | 10↑ | no | | 4.3↑ | no | 4↓ | Predicted gene 10050, is a protein-coding gene in the species Mus musculus (NCBI). |

Supplementary Table 2 The top 10 significantly downregulated DE genes in the high-dose NBXH group vs TB model group and their changes in other dose NBXH groups vs TB model group, JHW group vs TB model group, and TB model group vs normal group

| **GenBank accession** | **Gene**  **symbol** | **NBXH groups vs TB model group** | | | **JHW group vs. TB model group** | **TB model group vs. normal group** | **Annotation** |
| --- | --- | --- | --- | --- | --- | --- | --- |
|  |  | **High dose** | **Middle dose** | **Low dose** |  |  |  |
| NM_028025 | Mageb16 | 8↓ | 10↓ | 5↓ | no | no | Melanoma-associated antigen gene family member B16, is a key regulator of differentiation processes in embryonic stem cells via regulation of the expression of pluripotency factors[7]. It is suggested to play a role in cancer predisposition and to be associated with cancer risk[8]. |
| NM_021363 | Svs3a | 6↓ | 9↓ | 4↓ | no | 2↑ | Seminal vesicle secretory protein 3A, was predicted to enable protease binding activity and zinc ion binding activity (NCBI). |
| NM_007670 | Cdkn2b | 5↓ | 2↓ | no | no | 2↑ | Cyclin-dependent kinase inhibitor 2B, is a tumor suppressor gene that regulates the cell cycle by inhibiting the activity of cyclin-dependent kinase 4/6, and loss of its expression can cause the occurrence and development of various tumors[9]. |
| NM_001164118 | Serpinb6a | 5↓ | 3↓ | no | no | 5↑ | Serpin family B member 6a, inhibits cathepsin G (CatG) activity in mononuclear- and neutrophil-mediated immunoinflammatory responses, prevents apoptosis of immune cells, and promotes survival of immune cells[10]. |
| NM_001290660 | Cd302 | 5↓ | no | no | no | 15↑ | C-type lectin domain family 13 member A, is a recently discovered potential multifunctional C-type lectin receptor that may be involved in endocytosis, phagocytosis, cell adhesion, and migration processes, and participate in immune responses against viral and bacterial infections by regulating monocyte/macrophage function[11, 12]. |
| LF201819 | Ina | 4↓ | 5↓ | 4↓ | 3↓ | no | Internexin neuronal intermediate filament protein alpha, is involved in the formation and maintenance of neuronal morphogenesis and is a major component of the cytoskeleton in cerebellar granulosa cells of the adult central nervous system [13]. |
| NM_022420 | Gprc5b | 4↓ | 3↓ | no | no | no | G protein-coupled receptor class C group 5 member B, is an important regulatory factor for pancreatic islet function. Its high expression promotes apoptosis of β cells, which is related to type II diabetes; and its downregulating expression can increase insulin secretion and promote β cell survival[14, 15]. |
| NM_007664 | Cdh2 | 4↓ | 4↓ | 3↓ | no | 2↑ | Cadherin-2, is a calcium-dependent cell adhesion protein that maintains cell integrity and participates in a variety of cell signal transduction pathways. It is usually upregulated in various cancers and closely related to various types of cancers [16]. |
| NM_001034859 | Gm4841 | 4↓ | no | no | no | 24↑ | Interferon-inducible GTPase-like protein, predicted to enable GTPase activity, is involved in cellular response to interferon and defense response and is active in endoplasmic reticulum membrane (NCBI). |
| NM_030738 | Vmn1r65 | 4↓ | 3↓ | no | no | no | Vomeronasal 1 receptor 65, expresses an ultrasensitive G-protein coupled receptor only in the lamellar olfactory epithelium of amphibians and mainly in the vomeronasal organs of mammals[17]. |

Supplementary Table 3  The upregulated pathways in high-dose NBXH group vs TB model group and their changes in other dose NBXH groups vs TB model group, JHW group vs TB model group, and TB model group vs normal group

| **Pathway ID** | **Definition** | **NBXH groups vs TB model** **group** | | | **JHW group vs TB model group** | **TB model group vs normal group** |
| --- | --- | --- | --- | --- | --- | --- |
|  |  | **High dose** | **Low dose** | **Middle dose** |  |  |
| Mmu04650 | Natural killer cell-mediated cytotoxicity | 5.892759↑ | no | no | no | 3.120503↓ |
| Mmu05144 | Malaria | 3.742157↑ | no | no | no | 2.177646↓ |
| Mmu05200 | Pathways in cancer | 1.924996↑ | no | no | no | 3.249503↑ |
| Mmu04928 | Parathyroid hormone synthesis, secretion, and action | 1.738147↑ | no | no | no | 1.662952↑ |
| mmu05332 | Graft-versus-host disease | 1.697337↑ | no | no | no | 12.022379↑ |
| mmu04022 | cGMP-PKG signaling pathway | 1.643142↑ | no | 1.63565↑ | no | no |
| mmu00982 | Drug metabolism - cytochrome P450 | 1.543869↑ | no | no | no | no |
| mmu04512 | ECM-receptor interaction | 1.30915↑ | 2.771772↑ | 3.247701↑ | no | no |

Supplementary Table 4 The top 10 significantly downregulated pathways in the high-dose NBXH group vs TB model group and their changes in other dose NBXH groups vs TB model group, TB model group vs normal group, and JHW group vs TB model group

| **Pathway ID** | **Definition** | **NBXH** **groups vs TB model group** | | | | | **JHW group vs TB model group** | **TB model group vs normal group** |
| --- | --- | --- | --- | --- | --- | --- | --- | --- |
|  |  | **High dose** | **Low dose** | | | **Middle dose** |  |  |
| mmu04612 | Antigen processing and presentation | 4.628266↓ | | no | no | | no | 16.309057↑ |
| mmu05165 | Human papillomavirus infection | 4.628195↓ | | no | no | | no | 4.583199↑ |
| mmu05330 | Allograft rejection | 3.768181↓ | | no | no | | no | 12.022379↑ |
| mmu05332 | Graft-versus-host disease | 3.768181↓ | | no | no | | no | 12.022379↑ |
| mmu04940 | Type I diabetes mellitus | 3.511222↓ | | no | no | | no | 11.048553↑ |
| mmu04934 | Cushing syndrome | 3.503919↓ | | no | 1.748582↓ | | no | 2.248941↑ |
| mmu05166 | Human T-cell leukemia virus 1 infection | 3.350793↓ | | no | no | | no | 9.116658↑ |
| mmu05320 | Autoimmune thyroid disease | 3.222756↓ | | no | no | | no | 9.091123↑ |
| mmu04145 | Phagosome | 3.152111↓ | | no | no | | no | 20.442292↑ |
| mmu05416 | Viral myocarditis | 2.971572↓ | | no | no | | no | 9.909195↑ |

1. Kasem, E., T. Kurihara, and K. Tabuchi, *Neurexins and neuropsychiatric disorders.* Neurosci Res, 2018. **127**: p. 53-60.

2. Sun, H.T., et al., *FoxQ1 promotes glioma cells proliferation and migration by regulating NRXN3 expression.* PLoS One, 2013. **8**(1): p. e55693.

3. Wang, Y., et al., *The Role of YB1 in Renal Cell Carcinoma Cell Adhesion.* Int J Med Sci, 2018. **15**(12): p. 1304-1311.

4. Zhao, Y., et al., *ZNF582 hypermethylation promotes metastasis of nasopharyngeal carcinoma by regulating the transcription of adhesion molecules Nectin-3 and NRXN3.* Cancer Commun (Lond), 2020. **40**(12): p. 721-737.

5. Wang, J., et al., *KRT72 might serves as a prognostic biomarker for patients with prostate cancer.* Asian J Surg, 2023.

6. Fujii, T., et al., *Activation of mouse Otop3 proton channels by Zn(2).* Biochem Biophys Res Commun, 2023. **658**: p. 55-61.

7. Gaspar, J.A., et al., *Depletion of Mageb16 induces differentiation of pluripotent stem cells predominantly into mesodermal derivatives.* Sci Rep, 2017. **7**(1): p. 14285.

8. Juhari, W.K.W., et al., *Whole-Genome Profiles of Malay Colorectal Cancer Patients with Intact MMR Proteins.* Genes (Basel), 2021. **12**(9).

9. Hjazi, A., et al., *CDKN2B-AS1 as a novel therapeutic target in cancer: Mechanism and clinical perspective.* Biochem Pharmacol, 2023. **213**: p. 115627.

10. Burgener, S.S., et al., *Cathepsin G Inhibition by Serpinb1 and Serpinb6 Prevents Programmed Necrosis in Neutrophils and Monocytes and Reduces GSDMD-Driven Inflammation.* Cell Rep, 2019. **27**(12): p. 3646-3656 e5.

11. Lo, T.H., et al., *Characterization of the Expression and Function of the C-Type Lectin Receptor CD302 in Mice and Humans Reveals a Role in Dendritic Cell Migration.* J Immunol, 2016. **197**(3): p. 885-98.

12. Kato, M., et al., *The novel endocytic and phagocytic C-Type lectin receptor DCL-1/CD302 on macrophages is colocalized with F-actin, suggesting a role in cell adhesion and migration.* J Immunol, 2007. **179**(9): p. 6052-63.

13. Schult, D., et al., *Expression pattern of neuronal intermediate filament alpha-internexin in anterior pituitary gland and related tumors.* Pituitary, 2015. **18**(4): p. 465-73.

14. Soni, A., et al., *GPRC5B a putative glutamate-receptor candidate is negative modulator of insulin secretion.* Biochem Biophys Res Commun, 2013. **441**(3): p. 643-8.

15. Atanes, P., et al., *Identifying Signalling Pathways Regulated by GPRC5B in beta-Cells by CRISPR-Cas9-Mediated Genome Editing.* Cell Physiol Biochem, 2018. **45**(2): p. 656-666.

16. Chen, Q., J. Cai, and C. Jiang, *CDH2 expression is of prognostic significance in glioma and predicts the efficacy of temozolomide therapy in patients with glioblastoma.* Oncol Lett, 2018. **15**(5): p. 7415-7422.

17. Nakamuta, S., et al., *Type 1 vomeronasal receptor expression in juvenile and adult lungfish olfactory organ.* Zoological Lett, 2023. **9**(1): p. 6.
